# Environmental hazards from pollution of antibiotics and resistance-driving chemicals in an urban river network from Malawi

**DOI:** 10.1038/s44259-025-00149-5

**Published:** 2025-10-09

**Authors:** Derek Cocker, Taonga Mwapasa, Roman Grabic, Kateřina Grabicová, Andrea Vojs Staňová, Kondwani Chidziwisano, Adam P. Roberts, Tracy Morse, Nicholas A. Feasey, Andrew C. Singer

**Affiliations:** 1 Malawi-Liverpool Wellcome Program, Kamuzu University of Health Sciences, Blantyre, Malawi; 2 Department of Clinical Sciences, Liverpool School of Tropical Medicine, Liverpool, UK; 3 David Price Evans Infectious Disease and Global Health Group, University of Liverpool, Liverpool, UK; 4 Centre for Water, Sanitation, Health and Appropriate Technology Development (WASHTED), Polytechnic, University of Malawi, Blantyre, Malawi; 5 Faculty of Fisheries and Protection of Waters, University of South Bohemia in České Budějovice, South Bohemian Research Center of Aquaculture and Biodiversity of Hydrocenoses, Vodňany, Czech Republic; 6 Department of Analytical Chemistry, Faculty of Natural Sciences, Comenius University in Bratislava, Bratislava, Slovak Republic; 7 Department of Civil and Environmental Engineering, University of Strathclyde, Glasgow, UK; 8 Department of Tropical Disease Biology, Liverpool School of Tropical Medicine, Liverpool, UK; 9 The School of Medicine, University of St Andrews, St Andrews, UK; 10 UK Centre for Ecology & Hydrology, Wallingford, UK

**Keywords:** Antimicrobials, Environmental sciences

## Abstract

African communities have a high prevalence of antimicrobial-resistant bacterial carriage, alongside high levels of antibiotic usage and environmental pollution. Limited access to water, sanitation and hygiene infrastructure and wastewater treatment facilities enables the dissemination of resistant bacteria, antimicrobials and antibiotic resistance-driving chemicals (ARDCs) into local rivers. Few data exist quantifying the chemical drivers of antimicrobial resistance (AMR) in urban aquatic environments from African settings. In this longitudinal surveillance study, we investigated an urban river network in Blantyre, Malawi over a continuous 12-month period, identifying a broad-range of chemical pollutants, including antibiotics, common pharmaceuticals, agricultural and industrial chemicals and heavy metals. Antimicrobial concentrations were found at levels selective for AMR and ARDCs exhibited seasonal variations, indicating that deficient sanitation infrastructure and anthropogenic factors result in high antibiotic and ARDC levels entering the river systems, which serve as an important ecological niche for the acquisition, maintenance and transmission of AMR.

## Introduction

Antibiotics are primarily used in the treatment and prevention of disease in humans and animals, alongside their non-medical use as growth promoters within the animal sector^1,2^. Recent estimates from 2021 highlighted antibiotic resistance (AMR) is associated with 4.71 million human deaths annually^3^ and will lead to an estimated economic loss of US$100 trillion every year by 2050 if urgent action is not taken^4^. Global health inequities and limited access to reserve antibiotics mean that the greatest burden of AMR will be felt in low and middle-income countries (LMICs)^5,6^. In these settings, AMR poses an additional threat to the livestock sector and, thus, to the livelihoods of millions who raise animals for subsistence^7^.

The role of the environment as a reservoir for resistant pathogens and the linked importance of environmental pollution has increasingly become clear, with a growing evidence base for its relevance to human health^8,9^. As such, it is critical to adopt a One-Health approach that encompasses environmental, human and animal health, when considering interventions that tackle AMR on a global scale^10,11^. Around 40–90% of antibiotics consumed by humans and animals are excreted in an active form, which are subsequently dispersed as antibiotic residues into sewerage systems, groundwater and the wider riverine network^10,12^. In addition, other non-antibiotic chemicals including human pharmaceuticals, plant protection products (i.e., herbicides) and metals are commonly found as pollutants in groundwater, surface water and rivers^13^. The presence of both antibiotics and other antibiotic resistance-driving chemicals (ARDCs) (i.e., pharmaceuticals, plant protection products and metals) in aquatic environments, promotes horizontal gene transfer and alters microbial communities, contributing to the dissemination of antimicrobial-resistance genes (ARGs) and subsequently poses downstream risks to human, animal, and ecological health^10,13–15^. In certain settings, this is compounded by pollution from inadequate treatment of healthcare-associated, industrial, domestic, and agricultural waste, boosting the xenobiotic-derived resistome in the environment^16^.

Within African countries, there is often a lack of access to adequate water, sanitation and hygiene (WASH) infrastructure and functioning water treatment facilities, leading to ineffectual waste management^17–19^. This results in high levels of faecal contamination and antibiotic pollution within local river systems^18,20^. In these settings, open waters, such as rivers, are frequently used for domestic purposes^18^, adding to the risks of contamination with antibiotics, ARDC and faecal pathogens. The urban environment is of particular concern, with increasing urbanisation in African countries posing added health hazards via the pooling of domestic sewerage^20^. This is compounded by agricultural run-off from subsistence and small-scale farming, which permits the dispersal of antibiotics and ARDCs into the environment, further exacerbating the levels of environmental pollution^21,22^.

In addition, many African countries are prone to extreme seasonal changes in rainfall and temperature, with climate-associated AMR risks^23^. Increased rainfall and climatic factors exacerbate the risk of untreated sewage entering the environment, overwhelming the sewage network and promoting the survival and dissemination of human enteric pathogens^17^. Research on the distribution and ecological hazards posed by resistance-driving chemicals and AMR bacteria and genes in urban rivers from these settings is scarce, particularly in sub-Saharan Africa (sSA)^24^. Therefore, it is important to establish a baseline for the presence of antibiotic residues and ARDCs from waterways in LMICs and determine the AMR and ecological risks posed by the concentrations of antibiotic residues and ARDCs. This knowledge could be used to conduct hazard assessments for human and environmental exposure to AMR bacteria, genes and the resistance-driving chemicals themselves. Moreover, knowledge of the chemical state of rivers is required to gauge the success of future interventions and stewardship efforts aimed at reducing the AMR burden in Africa.

Here, we investigate the presence of and fluctuations in river water contamination with antibiotics, and ARDCs (including pharmaceuticals, plant protection products and metals), at two key sites within an urban riverine network of Blantyre, Malawi. Additionally, we evaluate seasonal variations in antibiotic concentrations and quantify ecological risks of AMR using predicted no-effect concentration (PNEC) thresholds that have been agreed by the AMR Industry Alliance^14,25^. PNEC thresholds are a commonly used approach to assess the risk posed by chemicals to aquatic systems, with target levels of antibiotics in rivers, above which, selection for AMR is expected to occur^14^.

## Results

**Fig. 1:**
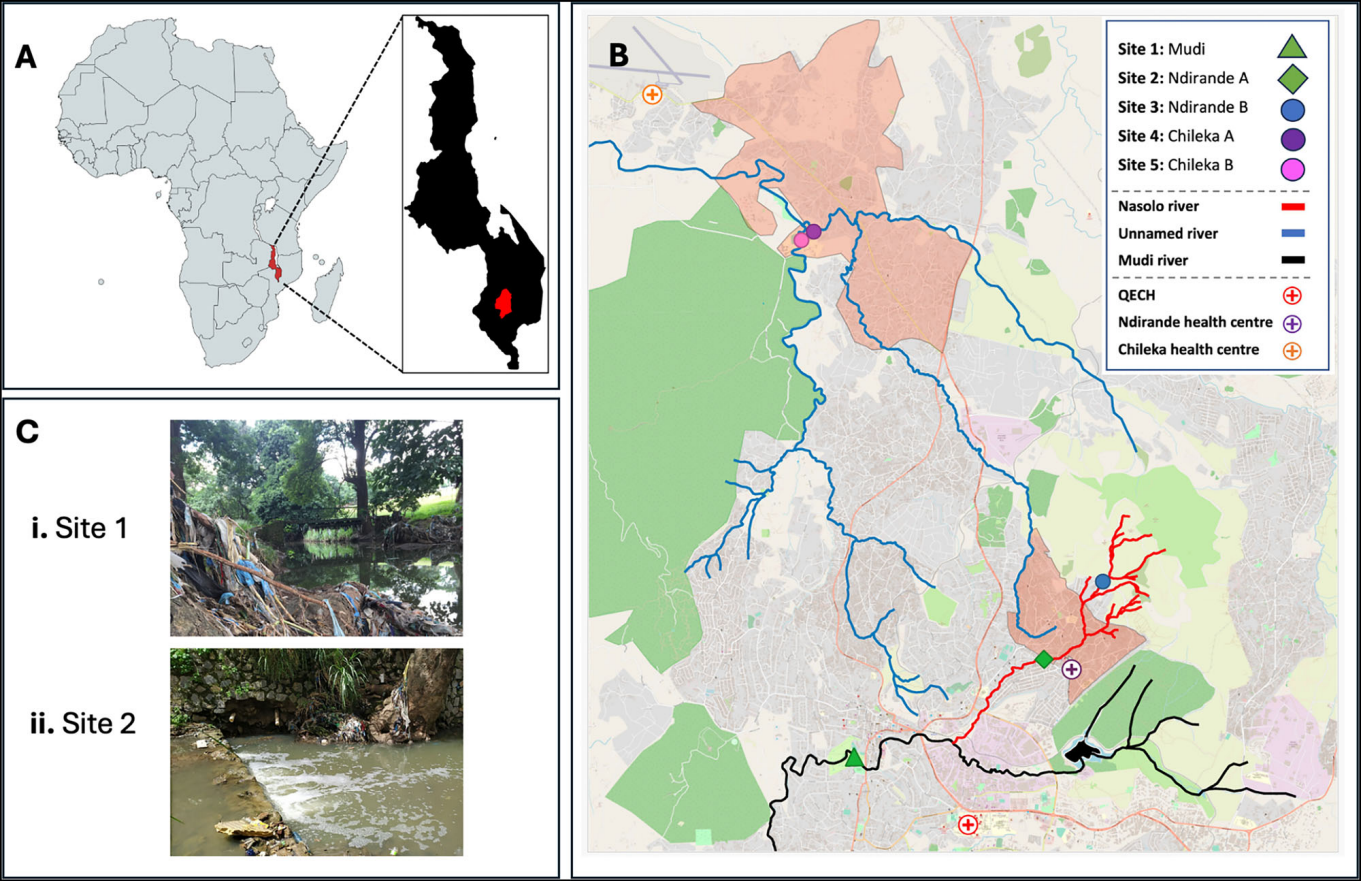
Description of the study setting, riverine networks and sampling sites. **A** Sampling was undertaken in Blantyre city in southern Malawi. **B** 5 river locations were screened during the pilot phase, including the Mudi river downstream of the urban centre (site 1), the Nasolo river, below (site 2) and above (site 3) the Ndirande township (shaded orange) and at two points along an unnamed river that flow through peri-urban communities on the outskirts of the city (sites 4 and 5 in Chileka, shaded orange). Sites 1 & 2 were enrolled into the longitudinal surveillance study, based on consistent year-round flow, logistics and safety profiling (appendix Table S1 and Figs. S11–3) and photos of these river sites at initiation are seen in **C** (Ci = Site 1, Cii = Site 2).

Five sites within an urban river system of Blantyre, Malawi were screened for acceptability to the populace and technical feasibility of sampling (Table S1) with 2 sites selected for longitudinal surveillance over an uninterrupted 12-month period between November 2020 to November 2021 (Fig. 1). Site 1 represented a river location downstream of the city centre/hospital and site 2 is a river location downstream of a dense urban community (Fig. 1b). Each river site underwent chemical sampling via Polar Organic Chemical Integrative Samplers (POCIS), which were submerged underwater and changed at weekly intervals. The average POCIS deployment time was 7.59 days (SD 2.64, range 6–22) for site 1 and 7.2 days (1.48, range 5–14) for site 2. A total of 96 POCIS were obtained over the 12 month period, and chemical analysis from these illustrated that both river sites were heavily contaminated with antibiotics and ARDCs, including medications intended for human use alongside products typically used in agriculture. In total, 38 antibiotics, 8 antiretrovirals, 2 antifungals, 3 antiparasitics, 49 non-antibiotic pharmaceuticals, 10 insecticides, 28 herbicides, 3 industrial chemicals, 8 fungicides and 25 heavy metals were recovered from rivers in urban communities (Figs. S1 and S2a–j).

### Presence of anti-infective agents and associated AMR risk

Antibiotics were found in all river samples that underwent analysis (100%, *n* = 96/96), including the presence of 12 sulphonamides (sulfadiazine, sulfamerazine, sulfamethazine, sulfamethoxazole, N4-acetylsulfamethoxazole, sulfamethoxine, sulfamethoxypyridine, sulfamoxole, sulfaphenazole, sulfapyridine, sulfaquinoxaline, sulfathiazole), 5 macrolides/lincosamide (azithromycin, clarithromycin, clindamycin, clindamycin sulfoxide, erythromycin), 6 β-lactams, including 4 cephalosporins (cloxacillin, penicillin-G, cefalexin, cefixime, cefuroxime, ceftriaxone), 9 fluoroquinolones (ciprofloxacin, difloxacin, enoxacin, enrofloxacin, flumequine, levofloxacin, lomefloxacin, norfloxacin, oxolinic acid) and members of 5 other antibiotic classes (chloramphenicol, metronidazole, rifampicin, trimethoprim, doxycycline), alongside 2 antifungal (clotrimazole, miconazole) and 1 antiparasitic (ornidazole) (Table 1). Non-targeted analysis further identified the presence of 8 antiretrovirals (abacavir, lamivudine, lopinavir, efavirenz, zidovudine, atazanavir, ritonavir and nevirapine), 2 antiparasitics used in malaria (sulfadoxine and pyrimethamine) and the tuberculosis antibiotic isoniazid (Fig. S1). 86.8% (*n* = 33/38) of antibiotics were recovered from both river locations, and 5 antibiotics (cefixime, doxycycline, enoxacin, penicillin-G, sulfamethoxypyridine) were identified at a single site (Fig. S2i-ii).

**Table 1:** Anthropogenic pollution of urban rivers with antibiotics, antifungals, antiprotozoals and other human-use medications





Medications recovered via continuous sampling of rivers in urban Blantyre over a 1-year period are presented with their mean (SD) concentrations (ng.POCIS^−1^.day^−1^). Differences in the concentrations of medications seen at sites (1-2) or between seasons (wet-dry) have been highlighted (Mann–Whitney (site) Wilcoxon test (season) *p* < 0.05 [blue = site, green = season]). The distribution of medication concentrations (ng.POCIS^−1^.day^−1^) are included in the Supplementary Information (Figs. S4e–g). Chemical metabolites = *. Chemicals where the limit of quantification ( < LOQ) was not reached as shown.

**Fig. 2:**
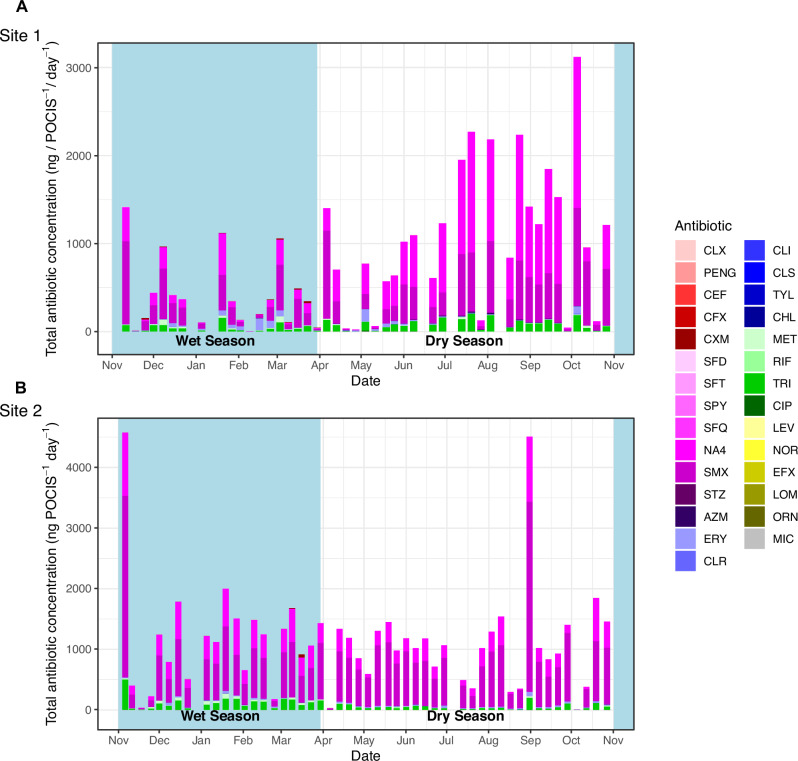
Spectrum of antibiotic and antifungal concentrations found in urban rivers over a 12 month period. Cumulative totals of antibiotic and antifungal concentrations (ng/ POCIS^−1^/ day^−1^) are shown for each POCIS recovered at both site 1 (**A**) and site 2 (**B**), illustrating the high and continuous presence of sulfamethoxazole (SMX), its metabolite (NA4) and trimethoprim (TRI) in urban rivers. (Wet season = blue, Dry season = white).

Antibiotics contributed 56.8% (Site 1: 41.96%, Site 2: 75.47%) of the total cumulative chemicals (ng.POCIS^−1^.day^−1^) recovered from rivers (Figs. S3 and S4g). The total concentrations of antibiotics recovered ranged from 0.22–22,000 ng.POCIS^−1^.day^−1^ (Fig. 2), and sulfamethoxazole (its metabolite N4-acetyl), trimethoprim, erythromycin and metronidazole were the dominant antibiotics found in river water, having both the highest detection frequency and mean (SD) concentrations (ng.POCIS^−1^.day^−1^) (Table 1, Fig. 2 and Fig. S5a, b). Here, for example, sulfamethoxazole was recovered at levels of 1400 (1500) ng.POCIS^−1^.day^−1^ at site 1, and 3100 (2100) ng.POCIS^−1^.day^−1^ at site 2 (Table 2). Additionally, macrolides and cephalosporins we consistently identified in urban rivers (Fig. S5a, b).

**Table 2:** Anthropogenic pollution of urban rivers with insecticides, herbicides, fungicides and industrial chemicals




Insecticides, herbicides, fungicides and industrial chemicals recovered via continuous sampling of rivers in urban Blantyre over a 1-year period are presented with their mean (SD) concentrations (ng.POCIS^−1^.day^−1^). Differences in the concentrations of chemicals seen at sites (1-2) or between seasons (wet-dry) have been highlighted (Mann–Whitney (site) Wilcoxon test (season) *p* < 0.05 [blue = site, green = season]). The distribution of chemical concentrations (ng.POCIS^−1^.day^−1^) are included in the Supplementary Information (Fig. S4a–d). Chemical metabolites = *. Chemicals where the limit of quantification ( < LOQ) was not reached as shown.

Variations in mean (SD) antibiotic concentrations depended on the antibiotic class and river site (Table 1, Fig. 2 and Fig. S4g). In the upstream dense urban community, we typically found higher levels of sulphonamides and tuberculosis therapies. In comparison, higher levels of macrolide and fluoroquinolones were found in the city centre downstream of the local hospital (Table 2, Fig. 2 and Fig. S4g). Unsurprisingly, sulfamethoxazole, its metabolite N4-acetyl, and trimethoprim were closely associated (Fig. S6), reflecting their presence in the antibiotic therapy co-trimoxazole and its use in the HIV programme as co-trimoxazole preventative therapy (CPT). Similarly, co-trimoxazole was associated with rifampicin, pointing toward the link between HIV and tuberculosis therapy.

There were fluctuations in the concentrations of antibiotics seen on a month-month basis at both sites, reflecting the seasonality of infectious disease and rainfall (Table 1, Fig. 2 and Fig. S5a, b), which in turn impacts upon the selection pressures within the riverine environment. However, fewer seasonal variations were seen in antibiotic presence or concentration than in other human pharmaceuticals or agricultural chemicals.

### Ecological antimicrobial risk quantification

**Fig. 3:**
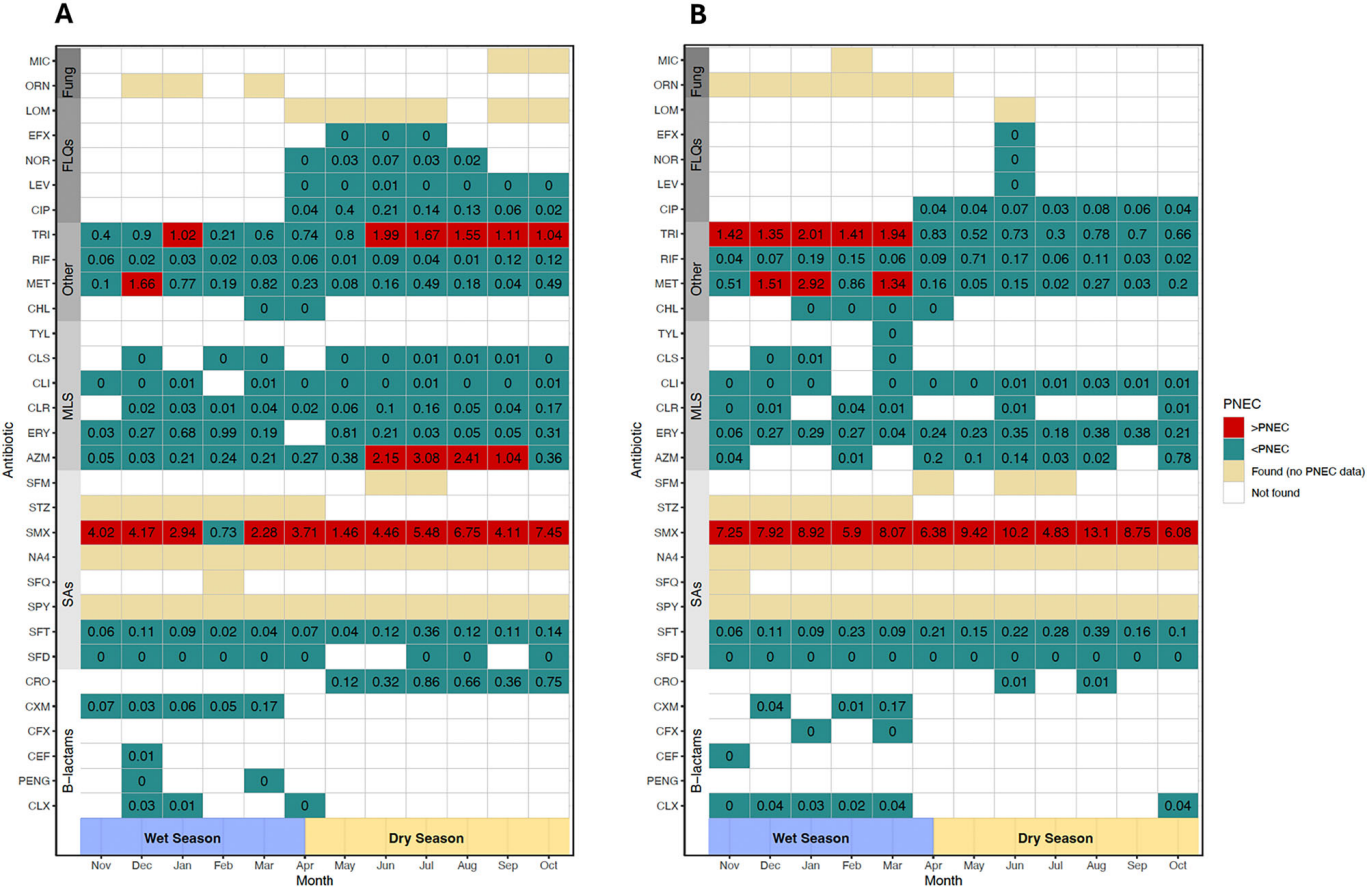
Temporal relationships in the recovery of antibiotics in river water, highlighting the continued presence of unsafe PNEC levels. Monthly trends in the presence and absence (white) of antibiotics are plotted over a 1-year period, spanning across the wet (blue) and dry (yellow) season at site 1 (**A**) and site 2 (**B**). Antibiotics are grouped by class, and stratified into safe (green, <PNEC) and unsafe (red, >PNEC) levels based on the concentrations identified. Values inside the cells describe the ratio of analyte: PNEC illustrating the levels of risk. A value of 0 denotes where an antibiotic was identified above the LOQ but below 0.01% of the agreed PNEC target. Cases where antibiotics are recovered, but there are no agreed PNEC definitions have also been highlighted (turquoise).

To determine whether antibiotic residues in urban rivers impacted on antimicrobial selection in the aquatic environment, monthly average concentrations were compared to published PNECs set out in the guidance from the AMR industry alliance; previously used in this manner in the UK (Fig. 3)^11,14,24^. Using this approach, the majority of individual antibiotic concentrations in urban rivers were below the PNEC threshold (80.95%, *n* = 17/21). However, sulfamethoxazole, trimethoprim, metronidazole and azithromycin were frequently recovered at levels above the upper limit of PNEC values. Here, trimethoprim and metronidazole were found at ~2 times the limit of PNECs, azithromycin was found at >3 times the PNEC and sulfamethoxazole was recovered all year round, and at levels that sometimes exceeded >10 times the PNEC threshold. Composite levels of macrolides showed additional levels of risk (Fig. S7a, b).

### Resistance-driving chemicals and medications

**Fig. 4:**
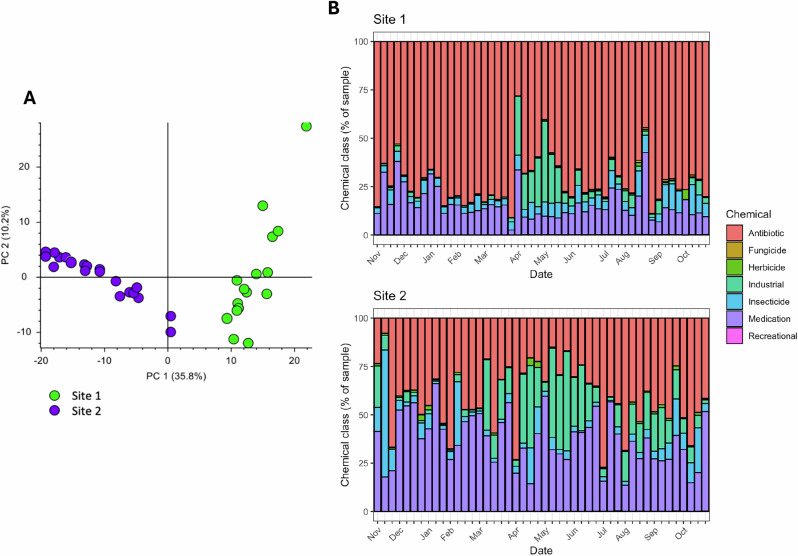
Composition of chemical pollutants found within urban rivers. **A** A principal component analysis of the spectrum of chemical compounds identified via non-targeted analysis of all POCIS obtained from site 1 and site 2 illustrating site-based differences. **B** The spatiotemporal variations in chemical compounds found at each site over a 1 year period. These are presented as the percentage (%) of the total POCIS sample chemical concentration normalised to sampling time (ng/POCIS^−1^/day^−1^), and stratified by chemical class.

Spatiotemporal variations in ARDCs were found in urban rivers, with insecticides, herbicides and fungicides exhibiting fluctuating levels throughout the year (Table 2 and Fig. 4), in contrast to antibiotics and human medications, which were often seen at consistently elevated levels (Table 1, Fig. 4 and Figs. S8a, b, S5a, b). Principle Component Analysis (PCA) highlighted that chemical composition differed substantially between sites (Fig. 4), likely reflecting differences in the geography upstream of the rivers (light industry and tertiary hospital effluent vs dense conurbation and agricultural land) (Fig. S1).

Overall, there were high levels of river contamination with chemicals used in agricultural and industrial practices (Table 2). 80.4% (*n* = 37/46) of analytes were recovered from both rivers, with a detection frequency of 90.0% (*n* = 9/10) for insecticides, 75.0% (*n* = 21/28) for herbicides and 87.5% (*n* = 7/8) for fungicides at both sites, and the minority found at a single location only (Fig. S2a–f). The mean (SD) analyte concentration normalized to sampling time (ng.POCIS^−1^.day^−1^) varied by location (Table 2 and Fig. S4a–d), with industrial chemicals and herbicides found at higher levels in the city centre (site 1) and neonicotinoid and organophosphate insecticides found at higher levels below the urban conurbation (site 2). Of note, DEET, chlorpyrifos, carbofuran and benzotriazole were found at particularly high concentrations (Table 2). Furthermore, a selection of ARDCs exhibited mean (SD) differences in concentrations depending on the season (wet vs. dry) (Table 2), with industrial chemicals found at proportionally higher concentrations at the end of the rainy season (Fig. 4), illustrating that seasonal changes in rainfall and local farming and agricultural practices lead to variations in the concentration of chemicals found in local rivers.

Medications used in human health were continuously recovered from urban rivers throughout the year (Figs. S8a, b and S9a, b), with 80.4% (*n* = 41/51) found at both sites and the rest recovered primarily from the site downstream of the tertiary hospital (Site 1, *n* = 9). Only sertraline and propranolol were found at levels that exceeded recognised PNEC or critical environmental concentration (CEC) targets (Fig. S10a, b). Mean (SD) concentrations (ng.POCIS^−1^.day^−1^) differed by site (Fig. S4e, f), with the highest levels of human pharmaceuticals frequently seen at Site 1 downstream of the local hospital (Table 2). Seasonal fluctuations existed (Table 1 and Figs. S9a, b), a notable example being antiepileptics, which were found at higher river levels during the dry season (Table 1 and Fig. S9a, b).

### Metals

**Fig. 5:**
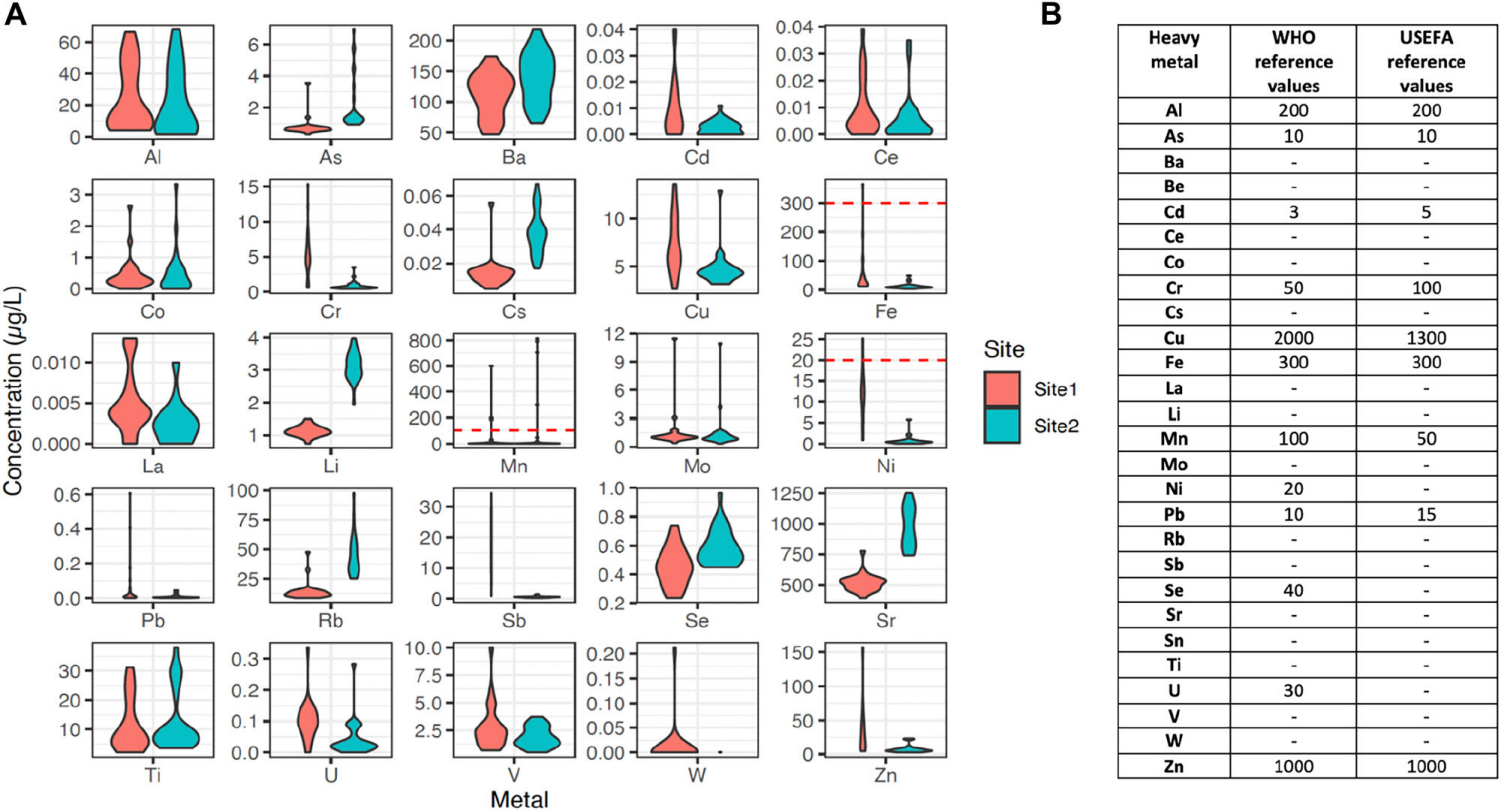
The presence and variations in heavy metals concentrations (µg/L) identified in urban waterways. **A** Violin plots showing the distribution of heavy metal concentrations (µg/L), stratified by site, including where acceptable levels have been exceeded by WHO reference standards [denoted by a dashed red line]. Results are obtained from 55 water samples (Site 1: *n* = 27, Site 2: *n* = 28) collected at weekly intervals between May 2021 and November 2021. **B** A list of accepted international reference standards for heavy metal concentrations (WHO/ USEFA).

River water was collected via 30 ml grab samples, undertaken at weekly intervals over a 6-month period, to evaluate the presence of metals. From the 55 water samples (Site 1: *n* = 27, Site 2: *n* = 28) obtained, chemical analysis illustrated that 25 different metals were repeatedly found in the rivers, with only 2 metals below the limit of quantification across all sites (Be and Sn) (Table S2). Metal concentrations (µg/L) varied by site and element, with median concentrations of Cu, Cr, Fe, Ni, Sb and Zn shown to be higher in the central urban river system downstream of the city centre (Fig. 5, site 1) and metal concentrations of As, Li, Rb and Sr higher in the river systems downstream of the dense urban conurbation (Fig. 5, site 2). Whilst none of the median (IQR) concentrations exceeded recognised World Health Organisation (WHO) or United States Environmental Protection Agency (USEPA) water quality standards^26^, isolated high levels of Ni ( > 20 µg/L), Mg ( > 100 µg/L) and Fe ( > 300 µg/L) were recorded in excess of these levels (Fig. 5 and Table S2).

## Discussion

In this study undertaken in an urban waterway from a large African city, we highlight that antibiotics are consistently recovered, including at levels that exceed PNECs, alongside the continued presence of ARDCs and heavy metals above reference limits, posing onward risks to human, animal, and ecological health. Antibiotic and ARDC pollution into freshwater systems creates the conditions for the maintenance of intrinsic antimicrobial resistance, the selection for new resistance mutations and/or the acquisition of mobile genetic elements conferring AMR^24,27,28^. The presence of antibiotics, crop and industrial chemicals, and metals in the environment increases the selection rate for antibiotic resistance, thereby allowing the environment to form a key niche for the maintenance and evolution of AMR^10,21^.

Previous research in African surface waters has identified sulphonamides, such as sulfamethoxazole, as the most commonly recovered antibiotics^24,29^. In this study, sulfamethoxazole was the antibiotic found at the highest levels, followed by trimethoprim, erythromycin/azithromycin, metronidazole and rifampicin. Despite a growing trend in intensive farming practices and the use of antibiotics for growth promotion in Malawi^30^, this spectrum of antibiotics reflects those typically used locally in human health to treat a broad range of bacterial diseases^31^. In urban Blantyre, the estimated HIV prevalence is 14.2%^32^ and the estimated community tuberculosis prevalence is 150–189 per 100,000 adults^33^. Therefore, the use co-trimoxazole preventative therapy and tuberculosis therapy, whether as primary treatment, or preventative for people living with HIV, is common. Previous research from urban settings in Malawi has shown that co-trimoxazole and amoxicillin are the most frequently used antibiotics^34^. A large cross-sectional survey of 1051 urban household members that took place in Blantyre between April and July 2021 highlighted that co-trimoxazole was taken by 11% of community members within the preceding 6-months and was also the most commonly antibiotic taken at the time of survey (3.7%)^34^. A second household study of urban, peri-urban and rural Malawi undertaken between April 2019 and Dec 2020 identified that 15.2% of community members had received an antibiotic in the preceding 6 months, primarily limited to oral amoxicillin, co-trimoxazole and metronidazole^18^. Within local healthcare, a different spectrum of antibiotics is used. Here, recent data from the local hospital showed that Ceftriaxone is prescribed to 76% of unselected adult patients, with metronidazole (8.4%), amoxicillin (7.6%) and ciprofloxacin (5.2%) also commonly given^35^. However, human antibiotic usage in sSA is complex, and influenced by the massive burden of infectious disease, vulnerabilities of access and cost and other intrinsic health system constraints^31^. Within Malawi, these conditions lead to a narrow spectrum of oral antibiotics typically being used^18,30^, reflected in antibiotic prevalence in urban surface waters. While campaigns to optimise community antibiotic prescribing are ongoing, antibiotic prescription is a rational response in settings where the burden of infectious disease is large and diagnostics are few. Therefore, a priority focus should be on waste management improvements and environmental control of antibiotic dispersal, instead of reducing potentially life-saving antimicrobial therapy^36^.

AMR selection risks posed by antibiotics and medications can be assessed using PNECs, initially proposed for use as discharge limits from manufacturing facilities or via critical environmental concentrations^14,24^. When we compared these targets to putatitive antibiotic concentrations found in urban rivers, sulfamethoxazole, trimethoprim, metronidazole and azithromycin were consistently recovered above recommended PNEC limits over extended periods of time. Given the absence of antibiotic manufacturing plants or functioning WWTP upstream of the river sites, these results suggest that ineffectual waste management of human effluent leads to the widespread dissemination of antibiotics in the urban riverine environment and that antibiotics, ARDCs, and heavy metals may serve as an important driver for AMR bacteria and ARGs in urban sSA rivers^18,37^. Tighter regulation of ARDCs and protecting urban waterways from chemical contamination may positively impact human health and the ecosystem.

Widespread co-contamination of the urban environment with ARB is commonplace. Within Blantyre, extensive soil, surface water and environmental contamination with ESBL Enterobacteriaceae (ESBL-E) is found in high-density environments, linked to several key environmental exposure risks^38^. This is further exacerbated by a lack of awareness of the risks posed from environmental pathways including human-environmental interactions, especially via contaminated river water^39^. A large, contemporaneous One Health study undertaken in the dense urban conurbation directly upstream of our river sites found extensive ESBL-E contamination within the rivers, drains and local environments, alongside high levels of ESBL-E gut colonisation of animals and humans^18^. Here, rivers were the most frequently contaminated environment, with an ESBL-E prevalence of 66% overall, 74% in urban settings, emphasising the importance of the urban riverine network. Genomic analysis of ESBL *E. coli* isolates from human/animal stool and environmental sources in that study showed they cluster independently of ecological source^40^, and extremely close genomic relationships were identified between samples from ecological compartments indicating recent transmission from common sources. This highlights the significance of the shared environment in driving AMR, including local river systems. Taken with the results presented in this study that show high levels of antibiotic and ARDCs in the local river systems, this reaffirms that in Malawi, AMR solutions require a One Health approach^36^.

Climate change and socioeconomic factors have been shown to substantially contribute to the global AMR crisis, particularly within LMICs^23^. Previous research conducted in Blantyre found seasonal relationships between the wet season and human AMR colonisation^18^ and environmental AMR contamination^38^. The presence of ARDCs in sub-tropical rivers is also influenced by seasonal trends in rainfall^37,41^, as illustrated by our findings showing fluctuations in the recovery and concentration of antibiotics and ARDCs over time, including wet vs dry seasons. High levels of rainfall leads to widespread flooding, increased runoff from agricultural sites and overflowing of pit latrines into local rivers and groundwater, increasing exposure to pathogens, ARDCs and AMR risks^17,20,22,37^. The paucity of adequate sanitation infrastructure in urban settings intensifies the effects of climactic events, impacting fluctuations of antibiotics and ARDCs in effluent and the local river systems^23,37^. Improving sanitation facilities, universal access to water, sanitation and hygiene services and adherence to sustainable development strategies will be paramount to the future success of global AMR efforts^23^.

Metals have also been identified in urban sSA river systems via point prevalence studies^42^. However, longitudinal data in this study highlights that concentrations occasionally exceed putative selection thresholds, illustrating ecological risks from contamination with heavy metals fluctuates and continuous surveillance and intervention are required. Additionally, agrochemicals, including insecticides, herbicides, and fungicides that are frequently used by households and subsistence farmers in Africa^43^ were detected and regularly recovered throughout the year. Whilst internationally agreed AMR thresholds in surface waters do not exist for these chemicals, their role is widely reported to influence selection pressures on bacteria in the aquatic environment via similar mechanisms to antibiotics, making the putative thresholds for selection (PNECs) conservative estimates of the actual minimum selective concentration^24,44,45^.

Many factors can impact the fate of antibiotics and ARDCs in the aquatic environment, including photodegradation, biodegradation and river flow rates. If these factors fluctuate throughout the year it could contribute to shifts in the recovery of some analytes, impacting the interpretation. A limitation of this study is that weekly sampling does not permit the assessment of hazard on a daily or hourly basis. Moreover, passive sampling does not reflect alterations in the concentrations on short timescales; as such, we are unaware of the extremes in concentration that some analytes might achieve, which will impact our interpretation of the prevalence of antimicrobial resistance genes in this setting. Furthermore, the absence of corresponding local field calibration of the POCIS does not permit the specific calculation of pollution in ng.L^−1^, given sampling parameters cannot be extrapolated from rivers elsewhere (i.e., Europe), due to different stream conditions in sub-Saharan Africa^46,47^. However, existing calibrations^47^ can be imposed on the POCIS from this study to gain insight into whether putative concentrations of the analytes are approaching established PNECs. This analysis is intended as a first glance and should be followed up in the future with research that considers continuous surveillance at a greater number of river sites over multiple seasons, alongside local field calibration of key river systems and the collection of linked bacterial, population-level and meteorological metadata, to determine the heterogeneity of urban pollution, distinguish anthropogenic pollution from natural baseline and to better evaluate the impact of ARDCs on driving AMR. Nevertheless, our study identified that in urban Malawian riverine environments, putative pollution levels with ARDCs and antibiotics exceeds PNECs considered safe for ecological health over extended periods. This finding is likely to be a consequence of inadequate WASH infrastructure in densely populated urban environments, human antimicrobial usage, and the local climate. Given the local river networks are a point of interaction in daily life, used for bathing, washing clothes, agricultural and animal practices^18^, the need for improvements in solid, human, and animal waste management are urgently required to impact the transmission and emergence of AMR, improve the health of residents, and enhance biodiversity. Improvements in waste management should be undertaken in parallel with enhanced environmental surveillance for ARDCs and AMR in urban river environments, the delivery of co-designed community education campaigns to highlight environmental risk and risk mitigation strategies alongside advocacy for a keener focus on the role of the environment in national AMR policies and regulatory frameworks. Given that ultimately, without widespread improvements to environmental health, we are unlikely to control AMR in these settings.

## Methods

### Pilot phase and site selection

This study was undertaken in Blantyre, southern Malawi, by the Drivers of Resistance in Uganda and Malawi (DRUM) consortium^48^. Blantyre has a population of ~830,000 people, is served by a single 1350-bed tertiary hospital, and has basic citywide sanitation infrastructure, with only one operational wastewater treatment plant (WWTP). An iterative approach to site selection was undertaken during a 9-month pilot phase (February 2020–October 2020). Initially, 5 sites were identified via transect walks undertaken in the urban and peri-urban districts alongside an area downstream of the city centre (Fig. 1 and Fig. S11). These included a geographical spread upstream and downstream of key urban river systems, with linkage to our previous community household study with extensive human, animal and environmental microbiological data^18^. Local chiefs and community leaders were surveyed for the acceptance of samplers and verbal permissions were granted. Where sites fell on private property, verbal agreements were drafted for placement of samplers prior to siting.

During the pilot phase, river water was purposively collected, and the utility of each site was assessed via a number of logistical and safety parameters. Three of the sites faced significant challenges from theft and the unsuccessful recovery of filters after a 7-day period. In particular, the furthest downstream river site had high fluctuations in rainfall during the wet season, leading to flash flooding and mechanical destruction of samplers, and 2 sites situated in dense urban environments where samplers were clearly visible, had high levels of theft. Given these logistical challenges and the requirement for continuous sampling over a 1-year period, two key rivers were selected for onward sampling (Figs. S12, 13). These sampling sites represented a river site downstream of the city centre/hospital (site 1) and a river site downstream of a dense urban community (site 2). Advice was sought from local leaders and community groups on how to best mitigate the issues identified during the pilot phase. In line with these conversations, mechanical and technical alterations to the recovery and placement of the filters were made (Table S1).

### Study design and sampling methods

Two urban river sites underwent uninterrupted sampling over a 12-month period (November 2020 to November 2021). POCIS [Nya Exposmeter AB, Trehorningen 34, SE-92266 Tavelsjo, Sweden, www.exposmeter.com] were submerged in the water at 20–100 cm depth at the fastest portion of the river and attached via metal wire to a stake on the riverbank, hidden from view. Each sampler was kept in place underwater throughout a week’s period at each river site. Then, at weekly intervals, the POCIS were recovered and replaced. At times of heavy rainfall where access posed a safety issue or during national holidays (i.e., Christmas), samplers were left in place for longer periods. On removal, the sampler cage was detached, and the membrane was washed with deionised water to remove any heavy soiling, before being placed in an aluminium foil bag, sealed, and transported to the laboratory within 2 h, whereupon it was stored at −80 °C. POCIS consists of a sorbent sandwiched between two polyethersulfone membranes, fixed into a porous metal cage (Fig. S14). The membrane allows for the passage of dissolved chemicals onto the sorbent, where they become sequestered^47^. Longitudinal metadata of river water parameters were contemporaneously collected.

In addition, river water sampling for heavy metals was completed at sites 1 & 2 (May 2021 and November 2021) at weekly intervals. Here, at each site, a 30 ml grab sample of river water was collected in a 30 ml universal container each week. Samples were transported to the local laboratory within 2 h and stored at ambient temperature in the dark. Subsequently, water samples were shipped at ambient temperature to the UK Centre for Ecology & Hydrology (United Kingdom) for metal analysis. POCIS filters were transported on dry ice to the University of South Bohemia (Czech Republic) for organic pollutant analysis.

### Chemical and heavy metal analysis

A suite of antimicrobials, medications, insecticides, herbicides, fungicides and metals were analysed based on evidence in the literature for their role in the selection or co-selection of antibiotic resistance genes and to examine a priori assumptions about antimicrobial use in Blantyre (Tables S3a–c). For metals, 10 ml of each sample was analysed by inductively coupled plasma mass spectrometry (Perkin Elmer Nexion 300D ICP-MS) screened against a panel of 27 metals (Table S3c) in line with standardised operating procedures at the UK Centre for Ecology & Hydrology. POCIS samplers were extracted using standard procedures described previously^46,49^. Targeted micropollutants analysis was performed using liquid chromatography coupled with tandem mass spectrometry (TSQ Quantiva mass spectrometer, Accela 1250 pump, both Thermo Fisher Scientific; PAL autosampler, CTC Switzerland)^47,50^. For quantification of analytes, the internal standard and matrix matching standard methods were used. Limits of quantification (LOQ) were calculated from the instrumental LOQ by correcting to the internal standard response, for the matrix effect, for internal standard response, and for the aliquot/volume of individual extracts^47^. Water concentrations of medications were calculated from POCIS adsorbed mass according to field calibration study data^47^. Due to the unknown flow of the river and absence of corresponding field calibration experiments, the concentration was presented as ng.POCIS^−1^ rather than ng.L^−1^. A value of ng.POCIS^−1^.day^−1^ was obtained by adjusting for the number of days between sample siting and recovery. However, to allow comparison of recovered analytes with published PNECs, we employed the previously published POCIS calibration^47^ to approximate the mean daily concentration per month for the respective analytes. The POCIS extracts were analysed using nontargeted LC-high resolution mass spectrometry (QExactive hybrid quadrupole-orbital trap mass spectrometer, Thermo Fisher Scientific) operated in combined full-scan/data independent modes^51^. The data were processed using Compound Discoverer 3.1 software to permit the identification of chemical compounds that were present but not included in the targeted analysis, including antiretrovirals, antibiotics, antifungals and antiprotozoals. PCA of nontargeted POCIS extracts data determined site-based differences in chemical compositions.

### Statistical analysis

Statistical analysis and graphic visualisations including ratios, means +/− standard deviation (SD), median +/− interquartile range (IQR), violin plots, Pearson’s coefficient matrix, PCA and PNEC tables were performed using R studio (Version 1.4.11)^52^. Sampling site maps were drawn using QGIS (Version 3.4). Analysis of the LOQ for concentrations includes the 1-year continuous dataset without pilot data. Differences in chemical concentrations found at locations and seasons were evaluated via Mann–Whitney and signed-rank Wilcoxon tests, respectively. PNEC values were obtained from international guidance and recent literature (Table S4a, b)^14,25^. The ratios of daily analyte mean per month / PNEC value were used to illustrate where antibiotics had exceeded accepted PNEC levels on a month-by month basis. CECs were taken for human pharmaceuticals where PNECs do not exist (Table S4b)^24,53^. The wet season was classified as samples obtained between November and April, and the dry season was classified as samples obtained between May and October.

### Ethical statement

This study was conducted within the DRUM consortium (MRC funded; MR/S004793/1) and as part of a personal fellowship (Wellcome Trust: 216221). The LC-MS analyses were performed at VVI CENAKVA Research Infrastructure (ID 90099, MEYS Czech Republic, 2019–2022), and nontargeted analyses were performed within grant No. 20-04676X funded by the Czech Science Foundation. Ethical approval was obtained for this study from the College of Medicine Research and Ethics Committee, Malawi (P.11/18/2541) and the Liverpool School of Tropical Medicine, UK (18-090). In addition, permissions were granted from village leaders, and support obtained from local community advisory groups. Sensitizations of study areas were conducted prior to study initiation.

## Supplementary information

NPJ_RiverWaterPaper_Appendix_(14_07_24)_reply_clean_copy_Final

## Data Availability

All relevant data are included in the manuscript and within the supplementary information.
