# Supplementary material for: Environmental hazards from pollution of antibiotics and resistance-driving chemicals in an urban river network from Malawi
Source: NPJ Antimicrob Resist. 2025 Oct 9;3:85. doi: 10.1038/s44259-025-00149-5 (PMC12511589; doi:10.1038/s44259-025-00149-5)
Supplement: Supplementary file 1 — NPJ_RiverWaterPaper_Appendix_(14_07_24)_reply_clean_copy_Final [file 44259_2025_149_MOESM1_ESM.pdf]

SUPPLIMENTARY INFORMATION

LIST OF TABLES AND FIGURES

Table S1. Strategy and logistical challenges at sampler sites.

Table S2. Presence of heavy metals and mean (SD) concentrations (µg/L).

Table S3a. List of antibiotics and their metabolites screened in river water samples.

Table S3b. List of insecticides, pesticides, herbicides, fungicides, industrial chemicals, recreational and common human use pharmaceuticals screened in river water samples.

Table S3c. List of metals screened in river water samples.

Table S4a. List of PNEC values (ng/L) adapted from the AMR industrial alliance discharge targets.

Table S4b. List of PNEC and CEC values (ng/L) used in Wilkinson JL et al

Figure S1. Non-targeted chemical analysis results.

Figure S2a. Presence and absence of insecticides and pesticides over a 1-year period at Site 1.

Figure S2b. Presence and absence of insecticides and pesticides over a 1-year period at Site 2.

Figure S2c. Presence and absence of herbicides over a 1-year period at Site 1.

Figure S2d. Presence and absence of herbicides over a 1-year period at Site 2.

Figure S2e. Presence and absence of fungicides over a 1-year period at Site 1.

Figure S2f. Presence and absence of fungicides over a 1-year period at Site 2.

Figure S2g. Presence and absence of medications over a 1-year period at Site 1, stratified by medication category (i.e. CNS, cardiovascular)

Figure S2h. Presence and absence of medications over a 1-year period at Site 2, stratified by medication category (i.e. CNS, cardiovascular)

Figure S2i. Presence and absence of antibiotics over a 1-year period at Site 1, stratified by antibiotic class.

Figure S2j. Presence and absence of antibiotics over a 1-year period at Site 2, stratified by antibiotic class.

Figure S3. Cumulative total of chemical compounds identified, stratified by site and coloured by chemical class.

FigureS4a. Violin plot of insecticide and pesticide concentrations (ng/POCIS^{-1}/day^{-1}) obtained from urban sites.

Figure S4b. Violin plot of herbicide concentrations (ng/POCIS^{-1}/day^{-1}) obtained from urban sites.

Figure S4c. Violin plot of fungicide concentrations (ng/POCIS^{-1}/day^{-1}) obtained from urban sites.

Figure S4d. Violin plot of industrial chemical concentrations (ng/POCIS^{-1}/day^{-1}) obtained from urban sites.

Figure S4e. Violin plot of recreational drug and analgesic medication concentrations (ng/POCIS^{-1}/day^{-1}) obtained from urban sites.

Figure S4f. Violin plot of common human-use pharmecutical concentrations (ng/POCIS^{-1}/day^{-1}) obtained from urban sites.

Figure S4g. Violin plot of antibiotic concentrations (ng/POCIS^{-1}/day^{-1}) obtained from urban sites.

Figure S5a. Spatiotemporal variations in antibiotic compositions at SITE 1, coloured by antibiotic class.

Figure S5b. Spatiotemporal variations in antibiotic compositions at SITE 2, coloured by antibiotic class.

Figure S6. Pearson's matrix of antibiotics in river water across all 5 study sites. Correlation coefficients are illustrated on a colour spectrum, with those in red and orange showing the highest degree of relationship.

Figure S7a. Temporal relationships in the recovery and concentrations of cumulative macrolide risk in river water, stratified by safe and unsafe PNEC levels for each site.

Figure S7b. Temporal relationships in the recovery and concentrations of cumulative fluoroquinolone risk in river water, stratified by safe and unsafe PNEC levels for each site.

Figure S8a . Spatiotemporal variations in medication compositions at SITE 1, presented as the percentage (%) of the total medication concentration (ng/POCIS^{-1}/day^{-1}), representing each medication.

Figure S8b . Spatiotemporal variations in medication compositions at SITE 2, presented as the percentage (%) of the total medication concentration (ng/POCIS^{-1}/day^{-1}), representing each medication.

Figure S9a. Cumulative total of medications identified from SITE 1, stratified by site and coloured by medication class.

Figure 9b. Cumulative total of medications identified from SITE 2, stratified by site and coloured by medication class.

Figure S10a. . Temporal relationships in the recovery and concentrations of medications in river water from SITE 1 stratified into safe and unsafe PNEC / CEC levels

Figure S10b. Temporal relationships in the recovery and concentrations of medications in river water from SITE 2 stratified into safe and unsafe PNEC / CEC levels.

Figure S11. Detailed maps of the riverine network of Blantyre, including (a) Blantyre city (b) Ndirande and (c) Chileka. DRUM study polygons have been demarcated in orange. Sampling sites have been geolocated (site 1: star, site 2: triangle, site 3: square, site 4: circle, site 5: diamond) alongside the key rivers (black = Mudi river, red = Nasolo river, blue = unnamed river).

Figure S12. Photos of the sampling sites at pilot study initiation. Local approvals and permissions were granted.

Figure S13. Seasonal changes in the rivers at sampling sites. Photos were taken during the pilot and continuous phase, after approvals and local permissions were granted.

Figure S14. Porous metal cage (a) sandwiches the PES membrane (b) allowing for environmental exposure while protecting the membrane integrity, which is attached to a metal wire that is secured to the river bank (c).

Table S1. LOGISTICAL CHALLENGES AT SAMPLER SITES AND MITIGATION STRATERGIES

Issue Identified	Solutions highlighted in consultation with local leaders and community groups	Alteration to the study processes
Mechanical destruction of the filter whilst situated in the river	Make the sampler less able to move around in the water.<br><br>Protect the POCIS filter with a cage or other device	The length of wire was reduced to ~20cm long to enable continuous submersal and limited movement.<br><br>The samplers were placed into a small surrounding cage to reduce the risk of mechanical destruction (pictured in Figure S4, adapted from "Instillation of POCIS samplers" by R Grabic, University of South Bohemia), and then submerged into the river
Theft of the filters when sited in the rivers.	Find places of least footfall that are ideally out of view from the public to site the filters<br><br>Alter the time of filter recovery to when less people are around<br><br>Utilise community champions to site and recover the filters, rather than study staff so as to not draw attention to where they are	Samplers were attached via a wire and secondary rope to metal posts drilled into the edge of the riverbank at points out of view from the public.<br><br>Collection and replacement of samplers were undertaken at times of reduced footfall, by members of the community known to the study team

Table S2. PRESENCE OF HEAVY METALS AND MEDIAN (IQR) CONCENTRATIONS (µg/L).

Metal	Site 1 median (IQR)	Site 2 median (IQR)	Metal	Site 1 median (IQR)	Site 2 median (IQR)
Al	11.4 (35.71)	10.72 (32.41)	Mo	1.05 (0.36)	0.95 (0.47)
As	0.68 (0.19)	1.35 (0.64)	Ni	11.7 (6.22)	0.4 (0.42)
Ba	116 (52.6)	139.5 (72.95)	Pb	0.01 (0.02)	< LOQ
Be	< LOQ	< LOQ	Rb	12.3 (4.35)	39.05 (19.82)
Cd	0.01 (0.01)	< LOQ	Sb	17.7 (15.23)	0.53 (0.19)
Ce	0.01 (0.01)	0.01 (0.01)	Se	0.43 (0.19)	0.56 (0.16)
Co	0.29 (0.34)	0.23 (0.57)	Sn	< LOQ	< LOQ
Cr	4.65 (2.53)	0.66 (0.37)	Sr	511 (73)	952 (291)
Cs	0.01 (0.01)	0.04 (0.01)	Ti	7.24 (11.02)	8.64 (8.39)
Cu	6.17 (3.91)	4.32 (1.02)	U	0.1 (0.06)	0.03 (0.03)
Fe	22.5 (30.95)	9.88 (5.25)	V	2.38 (1.61)	1.7 (1.48)
La	0.01 (0.00)	< LOQ	W	0.01 (0.04)	< LOQ
Li	1.09 (0.21)	3.17 (0.53)	Zn	25.42 (34.09)	5.56 (3.25)
Mn	2.23 (6.09)	1.88 (6.42)			

Table S3a. LIST OF ANTIBIOTICS AND THEIR METABOLITES SCREENED IN RIVER WATER SAMPLES

Antibiotic Class	Antibiotic Name	Acronym
β-lactams (β-Ls)	Amoxicillin	AMX
	Ampicillin	AMP
	Cloxacillin	CLX
	Flucloxacillin	FLX
	Penicillin G	PENG
	Penicillin V	PENV
	Cefalexin	CEF
	Cefixime	CFX
	Cefotaxime	CTX
	Cefuroxime	CXM
	Ceftriaxone	CRO
Quinolones (QNs)	Ciprofloxacin	CIP
	Difloxacin	DIF
	Enoxacin	ENX
	Enrofloxacin	EFX
	Flumequine	FLU
	Levofloxacin + Ofloxacin	LEV
	Lomefloxacin	LOM
	Norfloxacin	NOR
	Oxolinic acid	OXO
	Perfloxacin	PER
	Roxithromycin	ROX
MLS drugs *(MLS)*	Azithromycin	AZM
	Clarithromycin	CLR
	Clindamycin	CLI
	Clindamycin sulfoxide	CLS
	Erythromycin	ERY
	Tylosin	TYL
Sulphonamides *(SAs)*	Sulfadiazine	SFD
	Sulfamerazine	SFM
	Sulfamethazine	SFT
	Sulfamethizole	SFZ
	Sulfamethoxazole	SMX
	Sulfamethoxine	SMI
	Sulfamethoxypyridine	SMP
	Sulfamoxole	SML
	Sulfaphenazole	SPZ
	Sulfapyridine	SPY
	Sulfaquinoxaline	SFQ
	N1 Acetyl SMX	NA1
	N4 Acetyl SMX	NA4
	Sulfathiazole	STZ
Tetracyclines *(TCs)*	Chlortetracycline	CLT
	Doxycycline	DOX
	Oxytetracycline	OXY
	Tetracycline	TET

Other antibiotics<br>*(Other)*	Chloramphenicol<br>Florfenicol<br>Metronidazole<br>Rifampicin<br>Trimethoprim	CHL<br>FLO<br>MET<br>RIF<br>TRI
Antifungals and Antiprotozoals<br>*(Fung)*	Ornidazole	ORN
	Miconazole<br>Terbinafine<br>Clotrimazole<br>Ketoconazole	MIC<br>TER<br>CLZ<br>KET

Table S3b. LIST OF INSECTICIDES, HERBICIDES, FUNGICIDES, INDUSTRIAL CHEMICALS AND HUMAN USE PHARMACEUTICALS SCREENED IN RIVER WATER SAMPLES

Class	Chemicals and metabolites
**Insecticides and metabolites**	Carbofuran-3-hydroxy, Chlorantraniliprole, Chlorpyrifos, DEET, Diazinon, Dimethoate, Imidacloprid, Malathion,Methoxyfenozide, Pirimicarb, Pirimiphos_ethyl, Pirimiphos_methyl, Thiamethoxam, Warfarin
**Herbicides and metabolites**	1-(3,4-Dichlorophenyl)_urea, 2,4,5-Trichlorophenoxyacetic_acid, 2,4-Dichlorophenoxyacetic_acid, 2,4-Dichlorophenoxypropionic_acid, 3-chloro-4-methylaniline, 4-Isopropylaniline, Acetochlor, Acetochlor_ESA, Acetochlor_OA, Alachlor, Alachlor_ESA, Alachlor_OA, Ametryn, Anthranilic_acid_isopropylamide, Atraton,Atrazine, Atrazine_2-hydroxy, Atrazine_desethyl, Atrazine_desethyl-2-hydroxy, Atrazine_desethyl-desisopropyl, Atrazine_desisopropyl, Bensulfuron_methyl, Bentazone, Chloridazon, Chloridazon_desphenyl, Chloridazon_methyl_desphenyl, Chlorotoluron, Chlorotoluron_desmethyl, Clomazone, Cyanazine, Desmetryn, Dimethachlor, Dimethachlor_ESA, Dimethachlor_OA, Dimethenamid_ESA, Dimethenamid_OA, Diuron, Diuron_desmethyl, Fenuron, Florasulam, Fluazifop-p, Foramsulfuron, Hexazinone, Imazamethabenz_methyl, Imazamox, Ioxynil, Isoproturon, Isoproturon_didemethyl, Isoproturon_monodemethyl, Lenacil, Linuron, MCPA, MCPP, Metazachlor, Metazachlor_ESA, Metazachlor_OA, Methabenzthiazuron, Metobromuron, Metolachlor, Metolachlor_ESA, Metolachlor_OA, Metoxuron, Metribuzin, Metribuzin_desamino, Metsulfuron_methyl, Monolinuron, N-chloroacetyl-2,6-diethylaniline, Picloram, Prometryn, Propachlor, Propazine, Propazine_hydroxy, Sebuthylazine, Simazine, Simazine_hydroxy, Terbuthylazine, Terbuthylazine_desethyl, Terbuthylazine_desethyl-2-hydroxy, Terbuthylazine_hydroxy, Terbutryn,, Triallate
**Fungicides**	Azoxystrobin, Carbendazim, Cyproconazole, Dimethomorph, Epoxiconazole, Flusilazole, Metalaxyl, Metconazole, Propiconazole, Pyrimethanil, Tebuconazole, Triadimenol, Triticonazole
**Persistent industrial chemicals**	1H-benzotriazol, 1H-benzotriazol_(5/4)-methyl, 1H-benzotriazol_1-methyl
**Recreational and analgesic pharmaceuticals**	2-oxo-3-hydroxy-LSD, 6-acetylmophine, Amphetamine, Benzoylecgonine, Cannabinol, Catinone, Cocaine, Ketamine, MDA, MDEA, MDMA, Mephedrone, Metamphetamine, Methadone, Morphine, Norketamine, Oxycodone, THC-COOH
**Common human-use pharmaceuticals**	Alfuzosin, Alprazolam, Amitryptyline, Atenolol, Atorvastatin, Bezafibrate, Biperiden, Bisoprolol, Caffeine, Carbamazepine (CBZ), Dihydro CBZ, Epoxy CBZ, trans-dihydro-dihydroxy CBZ, Cetirizine, Cilazapril, Citalopram, N-desmethylcitalopram, Clemastine, Clomipramine, Clonazepam, Codeine, Diclofenac, Dicycloverine, Diltiazem, Diphenhydramine, Disopyramide, Donepezil, Eprosartan, Fenofibrate, Fexofenadine, Gabapentin, Glibenclamide, Glimepiride, Haloperidol, Iopromide, Irbesartan, Lamotrigine, Loperamide, Maprotiline, Meclizine, Memantine, Metoprolol, Metoprolol acid, Mianserin, Mirtazapine, Orphenadrine, Oseltamivir carboxylate, Oxazepam, Oxcarbazepine, Paroxetine, Pizotifen, Propranolol, Ropinirole, Rosuvastatin, Sertraline, Norsertraline, Sotalol, Sulfasalazine, Tamoxifen, Telmisartan, Terbutaline, Theophylline, Tramadol (TRM), N-desmethylTRM, O-desmethylTRM, Trazodone, Triamterene, Valsartan, Venlafaxine, O-Desmethylvenlafaxine, Verapamil, Vortioxetine

Table S3c. LIST OF METALS SCREENED IN RIVER WATER SAMPLES

Metal	Acronym	Metal	Acronym
Aluminium	Al	Molybdenum	Mo
Arsenic	As	Nickel	Ni
Barium	Ba	Lead	Pb
Beryllium	Be	Rubidium	Rb
Cadmium	Cd	Antimony	Sb
Cerium	Ce	Selenium	Se
Cobalt	Co	Tin	Sn
Chromium	Cr	Strontium	Sr
Caesium	Cs	Titanium	Ti
Copper	Cu	Uranium	U
Iron	Fe	Vanadium	V
Lanthanum	La	Tungsten	W
Lithium	Li	Zinc	Zn
Manganese	Mn		

Table S4a. LIST OF PNEC VALUES (ng/L) ADAPTED FROM THE AMR INDUSTRIAL ALLIANCE DISCHARGE TARGETS

Antibiotic	PNEC	Antibiotic	PNEC	Antibiotic	PNEC
Amikacin	16	Cloxacillin	0.13	Oxytetracycline	0.5
Amoxicillin	0.25	Colistin	2.0	Pefloxacin	8.0
Amphotericin B	0.02	Daptomycin	1.0	Phenoxymethylpenicillin	0.06
Ampicillin	0.25	Delamanid	0.03	Piperacillin	0.5
Anidulafungin	0.02	Doripenem	0.11	Polymixin B	0.06
Avibactam	200	Doxycycline	2.0	Retapamulin	0.06
Avilamycin	8.0	Enramycin	4.8	Rifampicin	0.06
Azithromycin	0.02	Enrofloxacin	0.06	Roxithromycin	1.0
Aztreonam	0.5	Ertapenem	0.13	Secnidazole	1.0
Bacitracin	8.0	Erythromycin	0.5	Sparfloxacin	0.06
Bedaquiline	0.08	Ethambutol	2.0	Spectinomycin	32
Benzylpenicillin	0.25	Faropenem	0.02	Spiramycin	0.5
Capreomycin	2.0	Fidaxomicin	0.02	Streptomycin	16
Cefaclor	0.50	Florfenicol	2.0	Sulbactam	16
Cefadroxil	2.0	Fluconazole	0.25	Sulfadiazine	13
Cefalonium	21	Flumequine	0.25	Sulfamethoxazole	0.6
Cefaloridine	4.0	Fosfomycin	2.0	Tedizolid	3.2
Cefalothin	2.0	Fusidic acid	0.5	Teicoplanin	0.5
Cefazolin	1.0	Gatifloxacin	0.13	Telithromycin	0.06
Cefdinir	0.25	Gemifloxacin	0.06	Tetracycline	1.0
Cefepime	0.5	Gentamicin	0.15	Thiamphenicol	1.0
Cefixime	0.06	Imipenem	0.13	Tiamulin	1.0
Cefoperazone	0.5	Isoniazid	0.13	Ticarcillin	8.0
Cefotaxime	0.1	Itraconazole	0.01	Tigecycline	1.0
Cefoxitin	8.0	Kanamycin	1.0	Tildipirosin	0.42
Cefpirome	0.06	Levofloxacin	0.25	Tilmicosin	1.0
Cefpodoxime	0.25	Lincomycin	0.81	Tobramycin	1.0

Cefquinome	1.6	Linezolid	6.7	Trimethoprim	0.5
Ceftaroline	0.06	Loracarbef	2.0	Trovafloxacin	0.03
Ceftazidime	0.5	Mecillinam	1.0	Tylosin	1.0
Ceftibuten	0.25	Meropenem	0.06	Vancomycin	8.0
Ceftiofur	0.06	Metronidazole	0.13	Viomycin	2.0
Ceftobiprole	0.23	Minocycline	1.0	Virginiamycin	2.0
Ceftolozane	1.9	Moxifloxacin	0.13		
Ceftriaxone	0.03	Mupirocin	0.25		
Cefuroxime	0.5	Nalidixic acid	16		
Cephalexin	0.08	Narasin	0.5		
Cephradine	N/A	Neomycin	0.03		
Chloramphenicol	8.0	Netilmicin	0.5		
Ciprofloxacin	0.06	Nitrofurantoin	64		
Clarithromycin	0.08	Norfloxacin	0.5		
Clinafloxacin	0.5	Ofloxacin	0.5		
Clindamycin	0.1	Oxacillin	1.0		

Table S4b. LIST OF PNEC AND CEC VALUES (ng/L) USED IN WILKINSON JL ET AL.

Medication	PNEC/CEC*	Medication	PNEC/CEC*	Medication	PNEC/CEC*
Amitriptyline*	48	Fexofenadine*	20222	Oxazepam*	30721
Amoxicillin	250	Fluconazole	25	Oxytetracycline	500
Atenolol	148000	Fluoxetine*	489	Paracetamol*	24000000
Carbamazepine	25000	Gabapentin	450000000	Pregabalin	100000
Cetirizine*	423061	Itraconazole	8	Propranolol	20
Cimetidine	176000	Ketoconazole	50	Ranitidine*	232954
Ciprofloxacin	60	Ketotifen*	12	Salbutamol*	27669
Citalopram*	141	Lidocaine*	466820	Sertraline*	51
Clarithromycin	250	Lincomycin	810	Sitagliptin	390000
Cloxacillin	130	Loratadine	0.56	Sulfadiazine	11210
Codeine*	26620	Metformin	1000000	Sulfamethoxazole	200
Diazepam	7800	Metronidazole	130	Tetracycline	1000
Diltiazem*	27884	Miconazole	200	Tramadol*	4799
Diphenhydramine*	2035	Naproxen	150000	Trimethoprim	500
Enrofloxacin	60	Nevirapine	12070	Venlafaxine*	6112
Erythromycin	500	Norethisterone*	486	Verapamil*	24

*Indicates predicted Critical Environmental Concentrations (CECs). CEC values have been obtained from those used in Wilkinson JL et al, which were originally predicted to represent the surface water concentrations in pharmaceuticals that would be expected to cause a pharmacological effect in fish.

Figure S1. NON-TARGETTED CHEMICAL ANALYSIS RESULTS. Additional chemical compounds identified through non-targeted analysis in site 1 and site 2 including the presence of antiretrovirals (ARV), antimalarials (AM) and anti-tuberculous medications (ATB) identified through non-targeted chemical analysis.

**Medication**	**Class**	**CAS No**	**Molecular formula**	**RT**	**Annot. DeltaMass [ppm]**	**Library used**	**Schmymanski level§**	**Dry season**		**Wet season**	
								Site 1	Site 2	Site 1	Site 2
**Abacavir**	**ARV**	136470-78-5	C14 H18 N6 O	6.04	1.24	mzCloud	2	95%	97%	93%	97%
**Nevirapine**	**ARV**	129618-40-2	C15 H14 N4 O	7.97	0.74	mzCloud	2	100%	100%	100%	97%
**Lamivudine**	**ARV**	131086-21-0	C8 H11 N3 O3 S	1.12	-0.87	massBanku EU	3	9%	0%	66%	93%
**Efavirenz**	**ARV**	154598-52-4	C14 H9 Cl F3 N O2	10.50	0.97	mzCloud	2	91%	93%	45%	97%
**Zidovudine**	**ARV**	30516-87-1	C10 H13 N5 O4	1.66	0.39	massBanku EU	3	27%	3%	69%	23%
**Lopinavir**	**ARV**	192725-17-0	C37 H48 N4 O5	10.82	0.86	mzCloud	2	95%	97%	97%	83%
**Sulfadoxine**	**AM**	2447-57-6	C12 H14 N4 O4 S	6.64	0.43	mzCloud	2	91%	97%	97%	97%
**Atazanavir**	**ARV**	198904-31-3	C38 H52 N6 O7	10.63	0.42	mzCloud	2	95%	97%	97%	87%
**Ritonavir**	**ARV**	155213-67-5	C37 H48 N6 O5 S2	10.66	1	mzCloud	2	73%	83%	17%	93%
**Pyrimethamine**	**AM**	58-14-0	C12 H13 Cl N4	7.48	1.49	massBanku EU	3	77%	69%	79%	23%
**Isoniazid**	**ATB**	54-85-3	C6 H7 N3 O	5.42	1.82	mzCloud	2	32%	14%	10%	0%

§ … Schymanski level according to a scale published in: Schymanski, E.L., Jeon, J., Gulde, R., Fenner, K., Ruff, M., Singer, H.P., Hollender, J., 2014. Identifying Small Molecules via High Resolution Mass Spectrometry: Communicating Confidence. Environmental Science & Technology 48, 2097-2098.

Figure S2a. PRESENCE AND ABSENCE OF INSECTICIDES OVER A 1-YEAR PERIOD AT SITE 1.

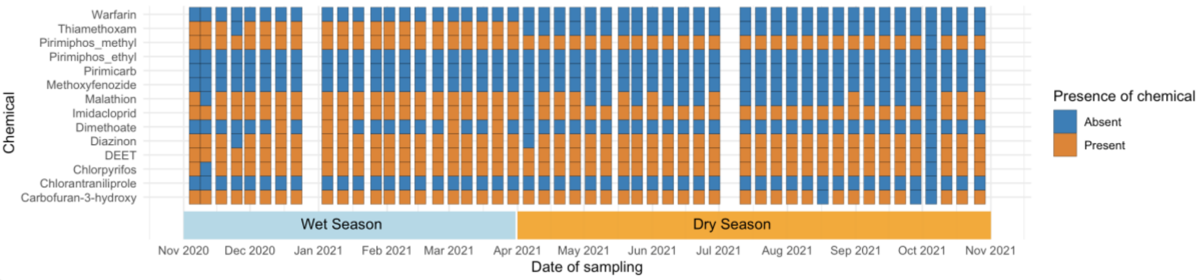

Figure S2b. PRESENCE AND ABSENCE OF INSECTICIDES OVER A 1-YEAR PERIOD AT SITE 2

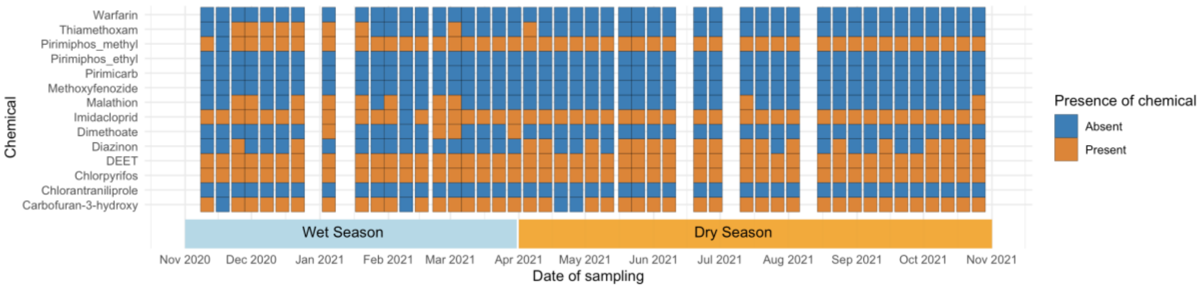

Figure S2c. PRESENCE AND ABSENCE OF HERBICIDES OVER A 1-YEAR PERIOD AT SITE 1

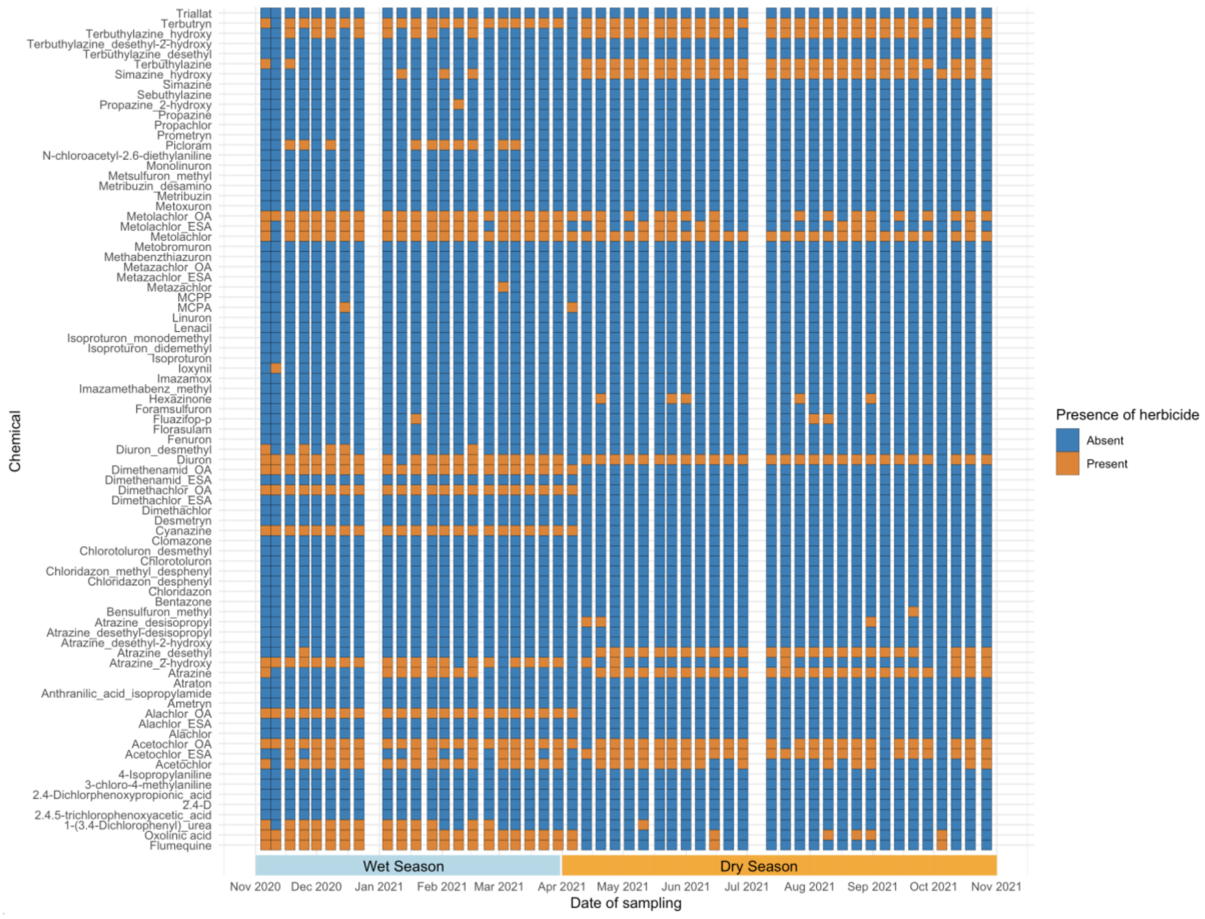

Figure S2d. PRESENCE AND ABSENCE OF HERBICIDES OVER A 1-YEAR PERIOD AT SITE 2

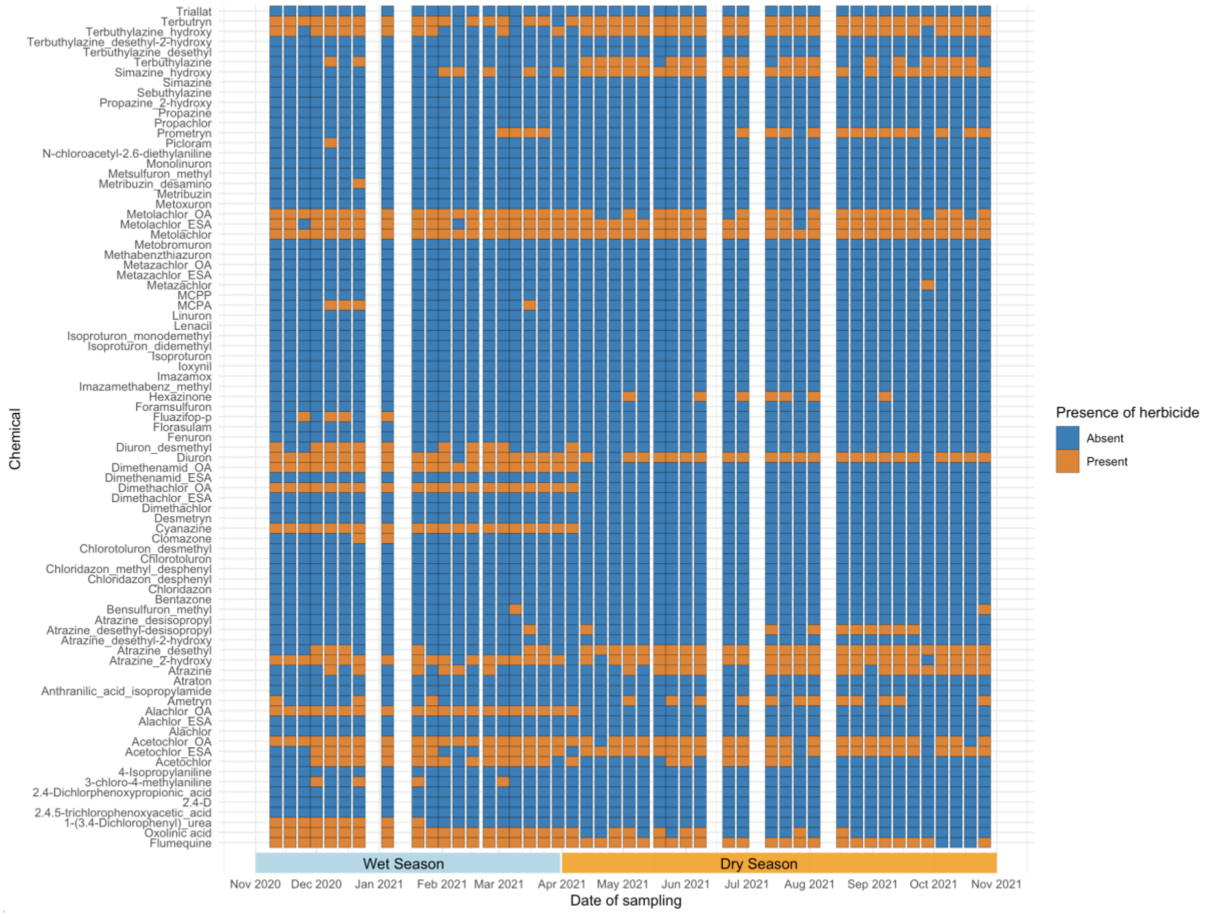

Figure S2e. PRESENCE AND ABSENCE OF FUNGICIDES OVER A 1-YEAR PERIOD AT SITE 1.

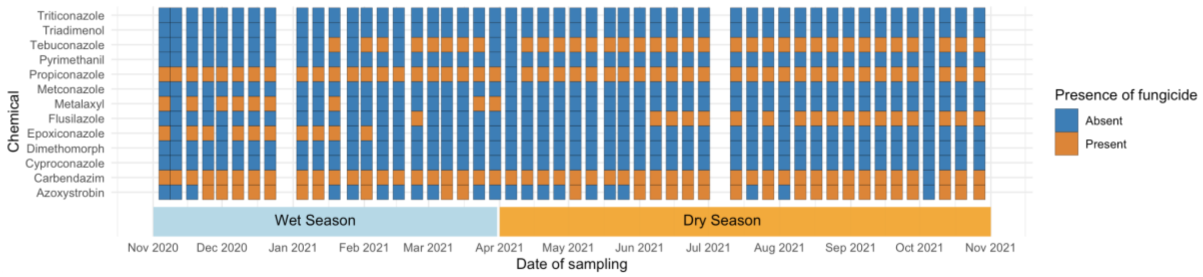

Figure S2f. PRESENCE AND ABSENCE OF FUNGICIDES OVER A 1-YEAR PERIOD AT SITE 2

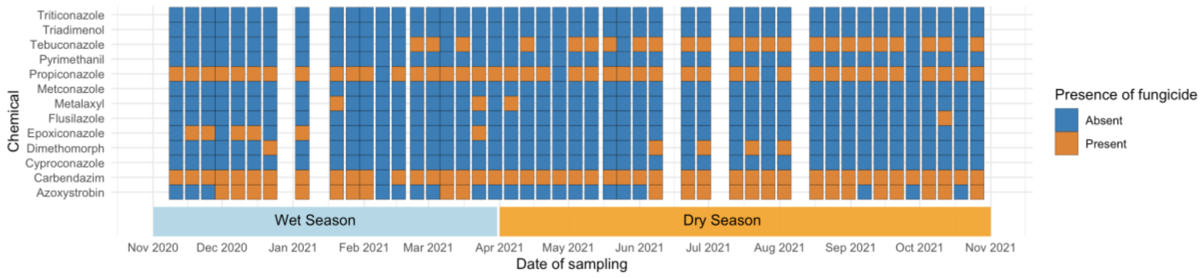

Figure S2g. PRESENCE AND ABSENCE OF MEDICATIONS OVER A 1-YEAR PERIOD AT SITE 1, STRATIFIED BY MEDICINE CATAGORY

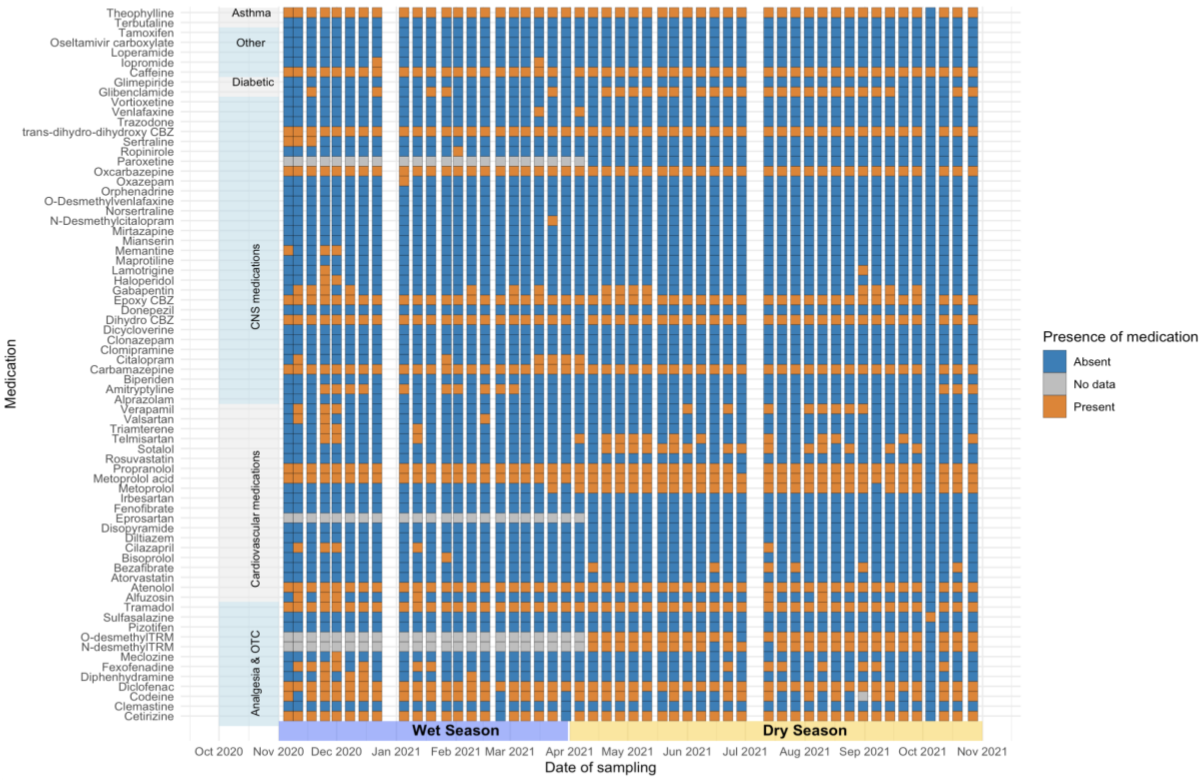

Figure S2h. PRESENCE AND ABSENCE OF MEDICATIONS OVER A 1-YEAR PERIOD AT SITE 2, STRATIFIED BY MEDICINE CATAGORY

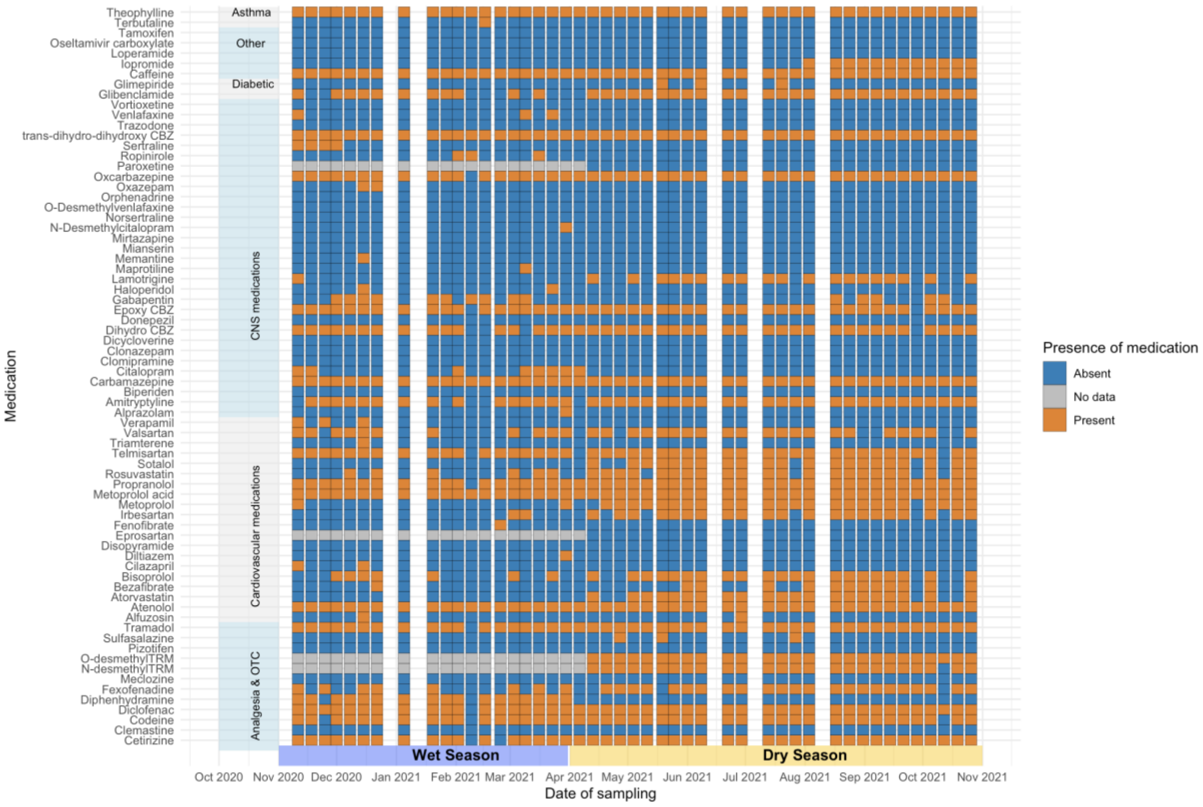

Figure S2i. PRESENCE AND ABSENCE OF ANTIMICROBIALS OVER A 1-YEAR PERIOD AT SITE 1, STRATIFIED BY ANTIBIOTIC CLASS.

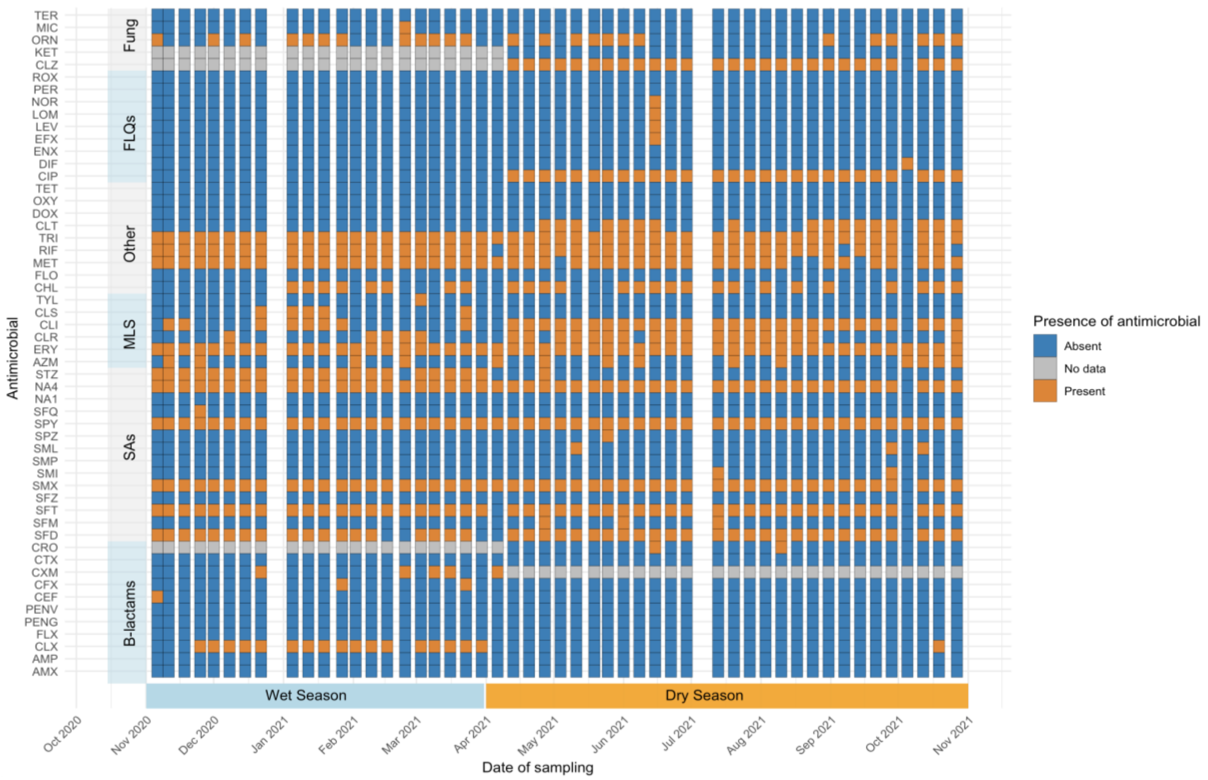

Figure S2j. PRESENCE AND ABSENCE OF ANTIMICROBIALS OVER A 1-YEAR PERIOD AT SITE 2, STRATIFIED BY ANTIBIOTIC CLASS.

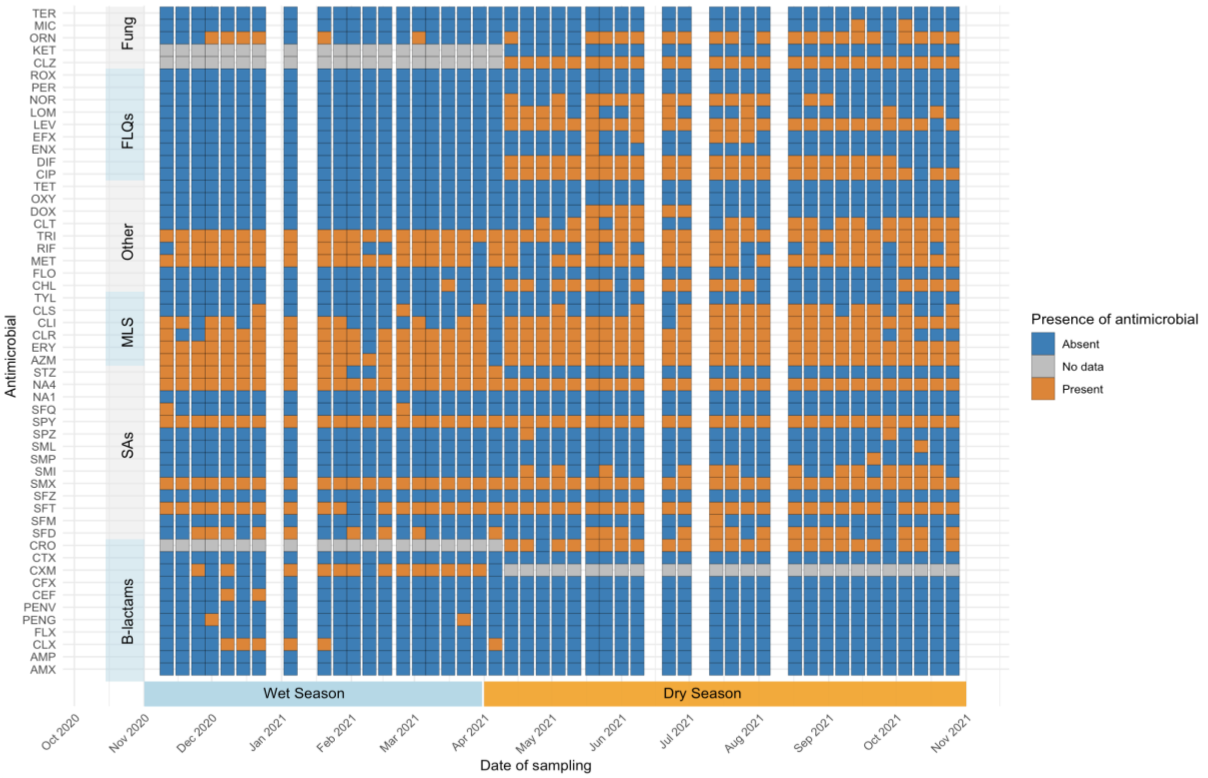

Figure S3. CUMULATIVE TOTAL OF CHEMICAL COMPOUNDS IDENTIFIED, STRATIFIED BY SITE AND COLOURED BY CHEMICAL CLASS.

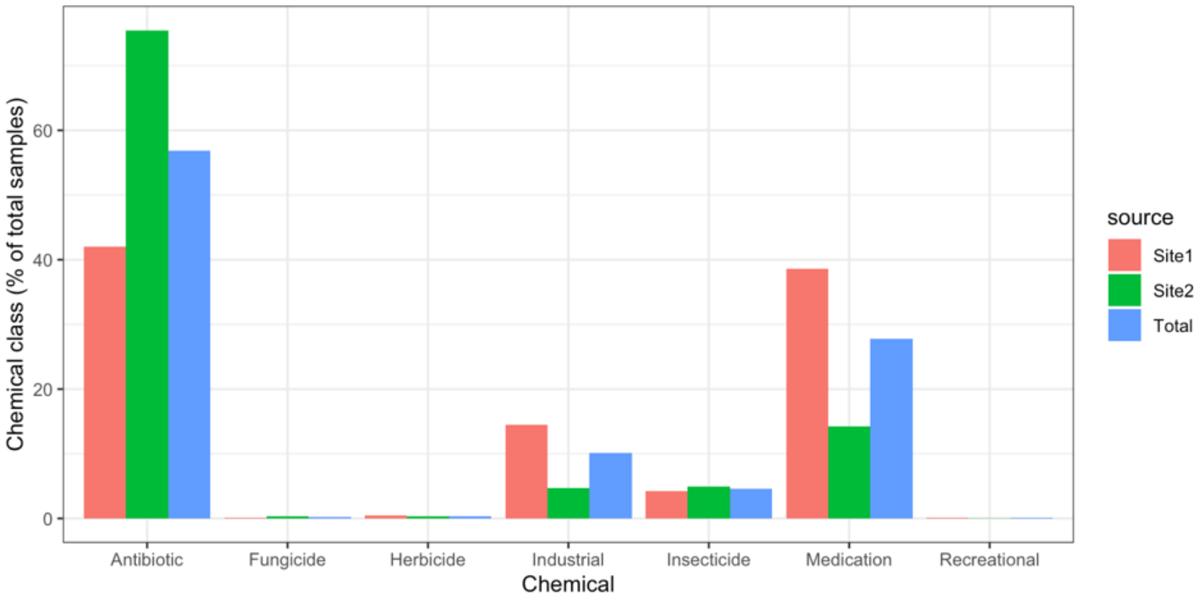

Figure S4a. VIOLIN PLOT OF INSECTICIDE CONCENTRATIONS (ng/POCIS^{-1}/day^{-1}) OBTAINED FROM URBAN SITES.

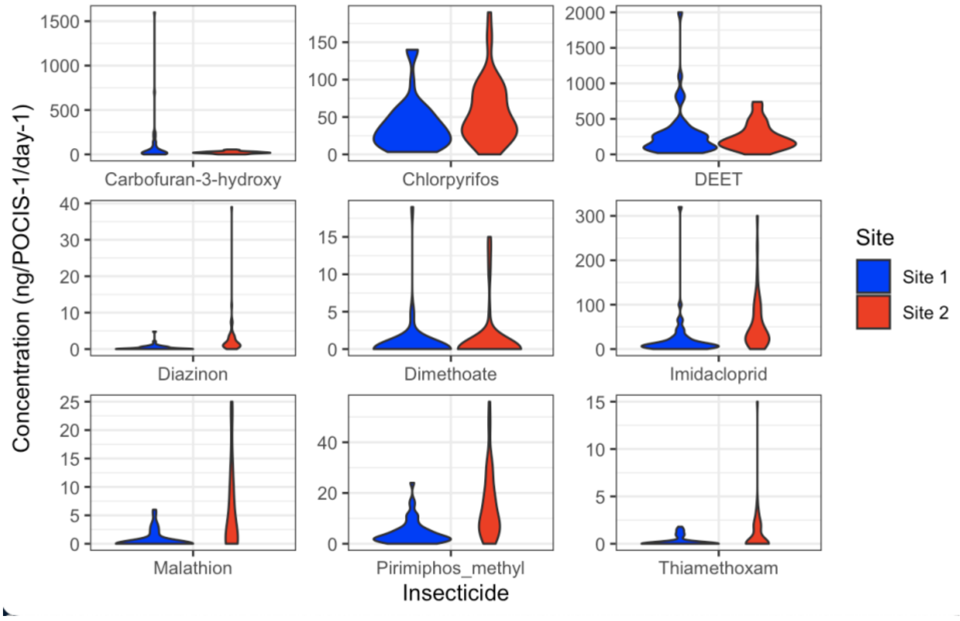

Figure S4b. VIOLIN PLOT OF HERBICIDE CONCENTRATIONS (ng/POCIS^{-1}/day^{-1}) OBTAINED FROM URBAN SITES.

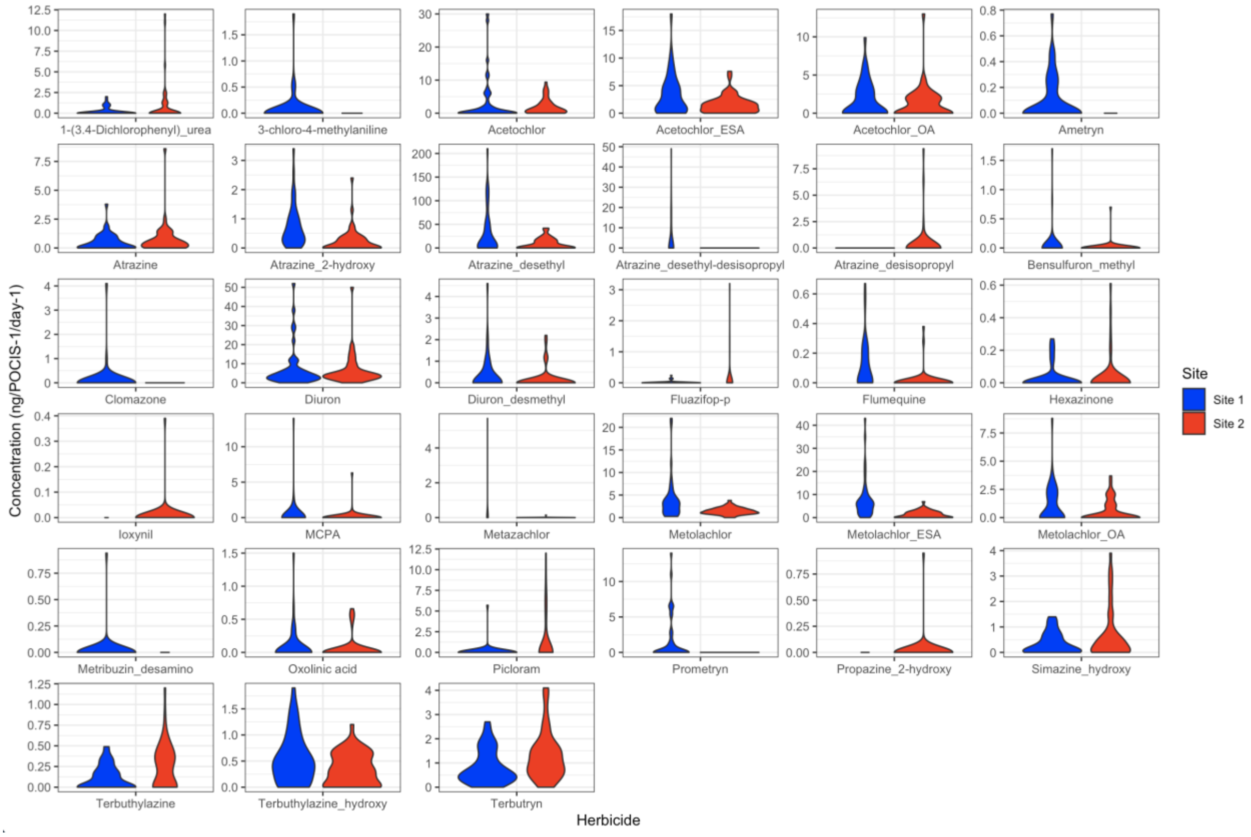

Figure S4c. VIOLIN PLOT OF FUNGICIDE CONCENTRATIONS (ng/POCIS^{-1}/day^{-1}) OBTAINED FROM URBAN SITES.

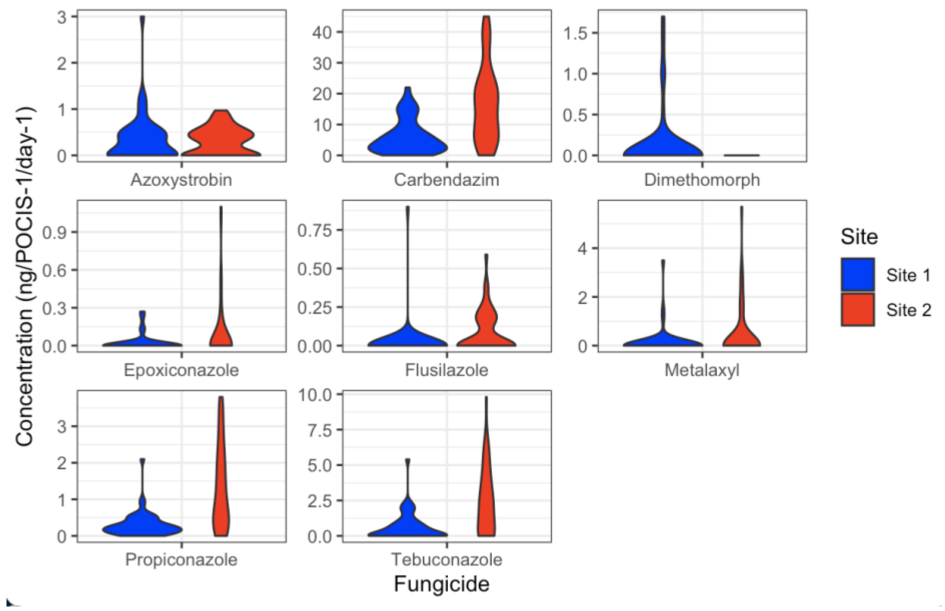

Figure S4d. VIOLIN PLOT OF INDUSTRIAL CHEMICAL CONCENTRATIONS (ng/POCIS^{-1}/day^{-1}) OBTAINED FROM URBAN SITES.

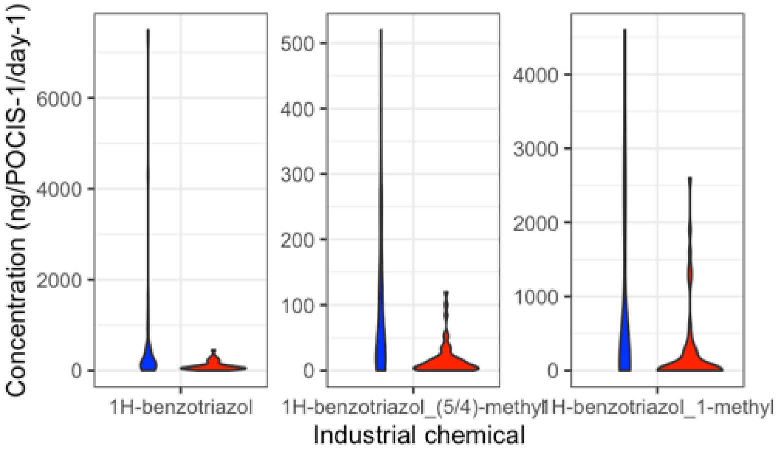

Figure S4e. VIOLIN PLOT OF RECREATIONAL DRUGS AND ANALGESIA CONCENTRATIONS (ng/POCIS^{-1}/day^{-1}) OBTAINED FROM URBAN SITES.

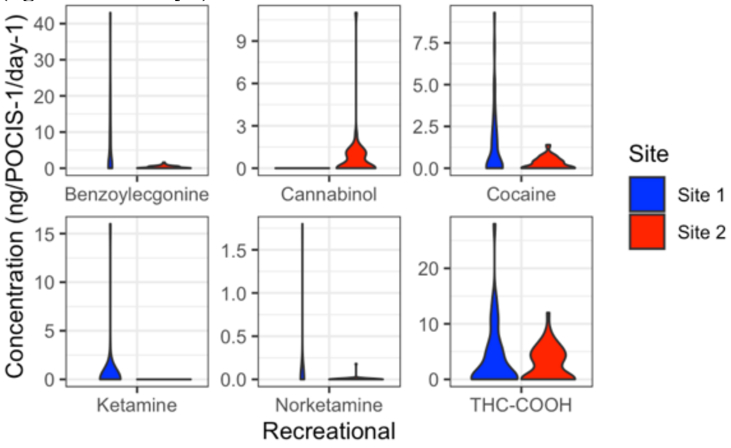

Figure S4f. VIOLIN PLOT OF HUMAN-USE PHARMACEUTICAL CONCENTRATIONS (ng/POCIS^{-1}/day^{-1}) OBTAINED FROM URBAN SITES.

CBZ – carbamazepine, TRM - tramadol

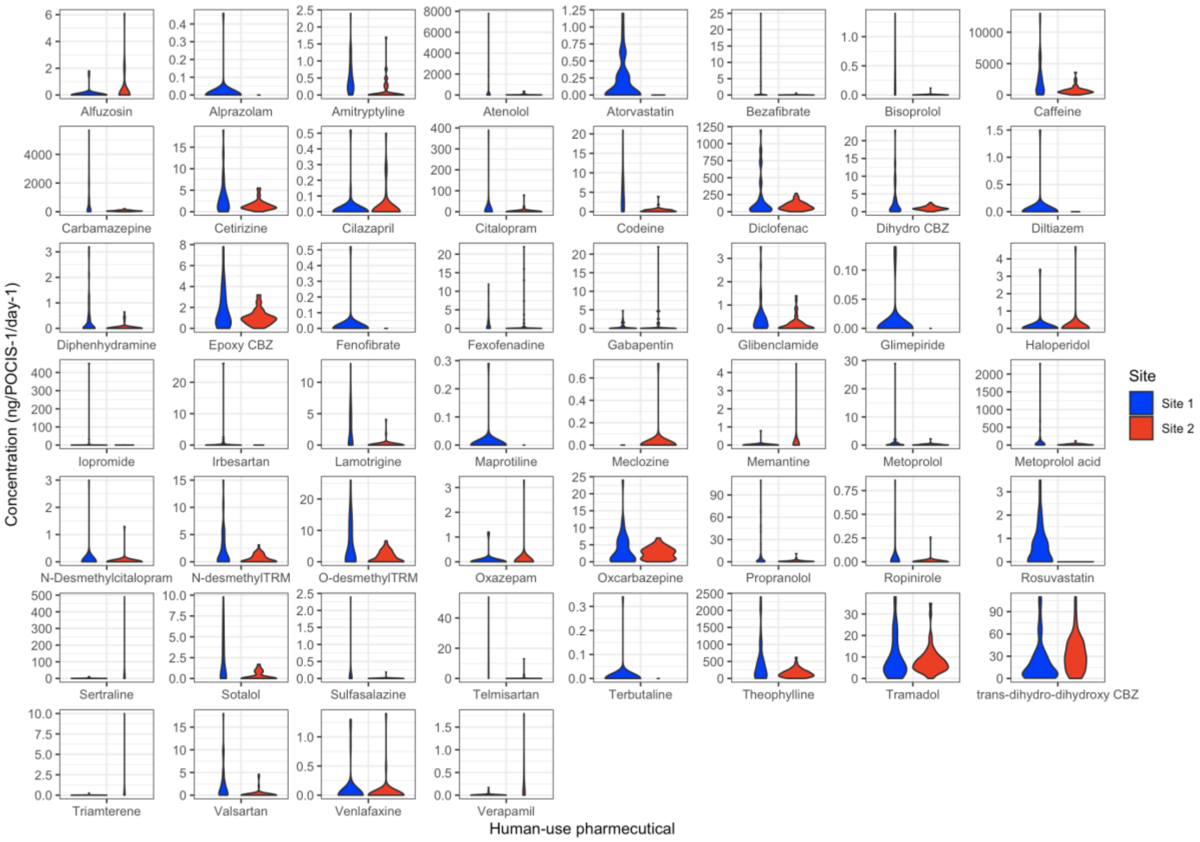

Figure S4g. VIOLIN PLOT OF ANTIMICROBIAL CONCENTRATIONS (ng/POCIS^{-1}/day^{-1}) OBTAINED FROM URBAN SITES.

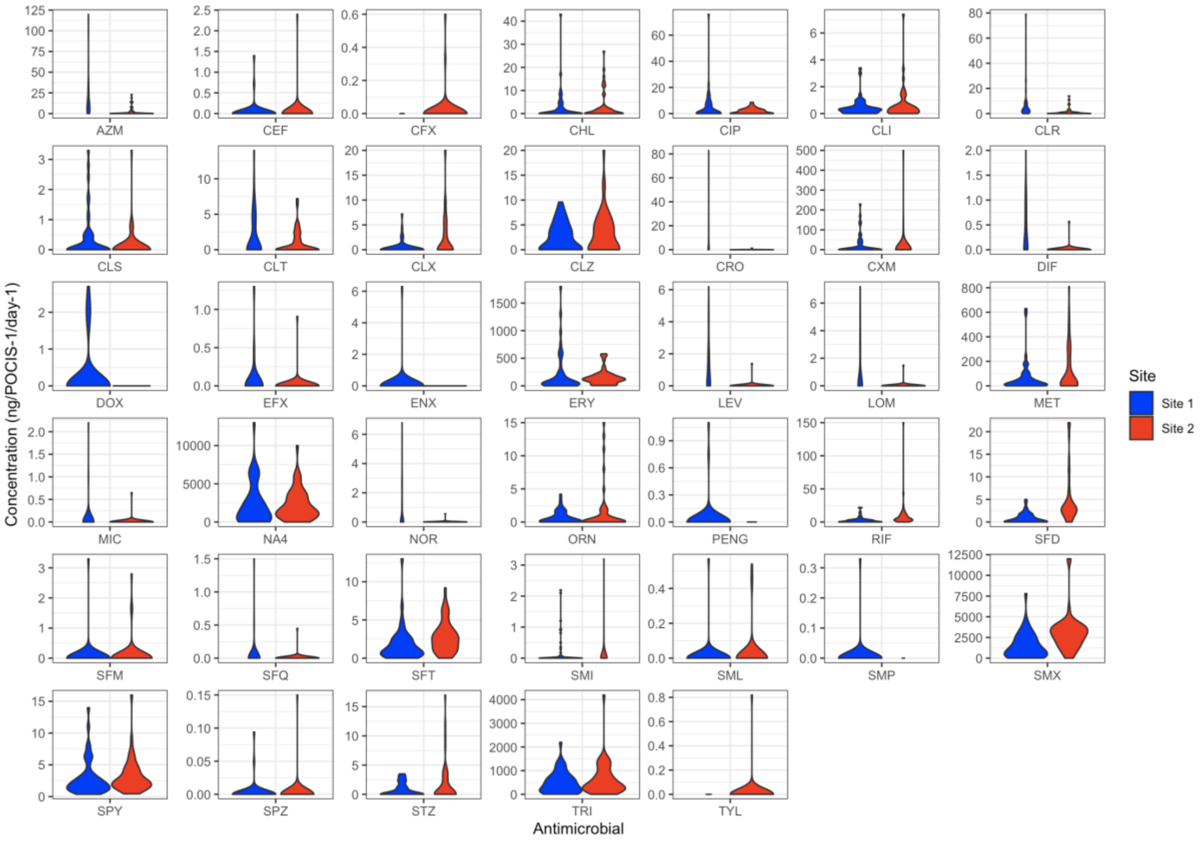

Figure S5a. SPATIOTEMPORAL VARIATIONS IN ANTIBIOTIC COMPOSITIONS AT SITE 1 OVER A 1-YEAR PERIOD. Presented as the percentage (%) of the total antibiotic concentration normalised to sampling time (ng/POCIS^{-1}/day^{-1}) coloured by antibiotic class.

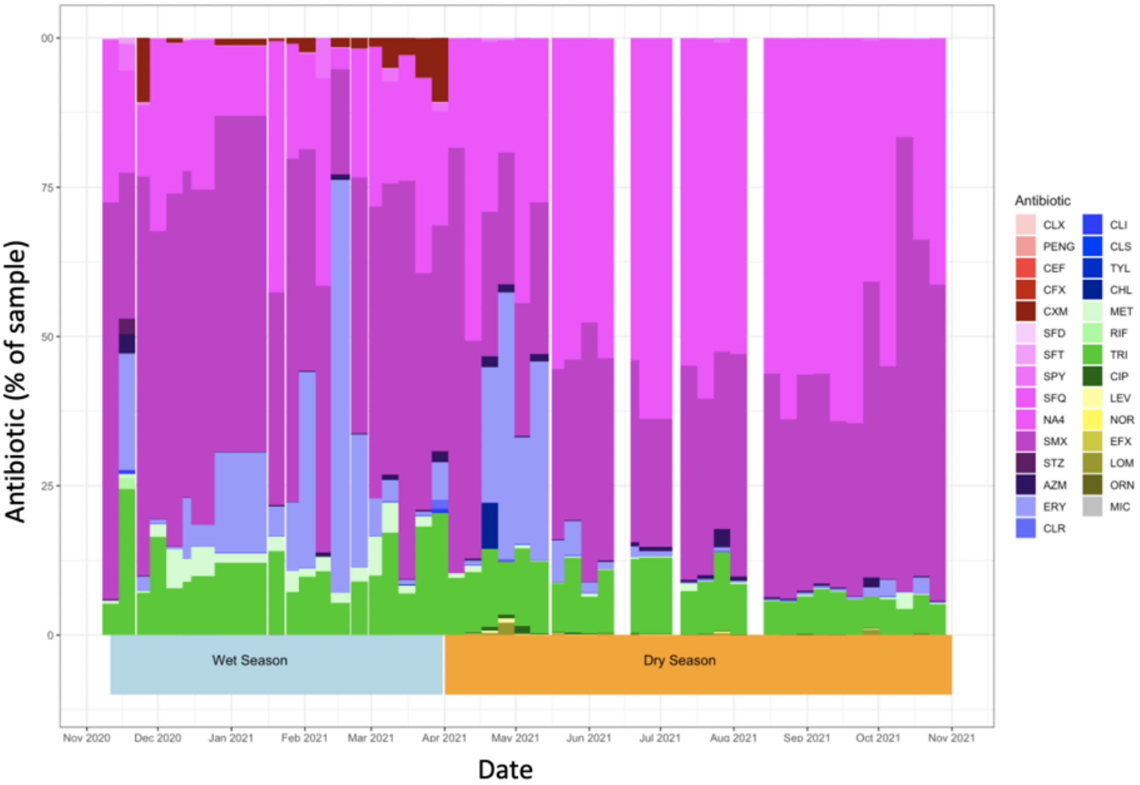

Figure S5b. SPATIOTEMPORAL VARIATIONS IN ANTIBIOTIC COMPOSITIONS AT SITE 2 OVER A 1-YEAR PERIOD. Presented as the percentage (%) of the total antibiotic concentration normalised to sampling time (ng/POCIS^{-1}/day^{-1}) coloured by antibiotic class.

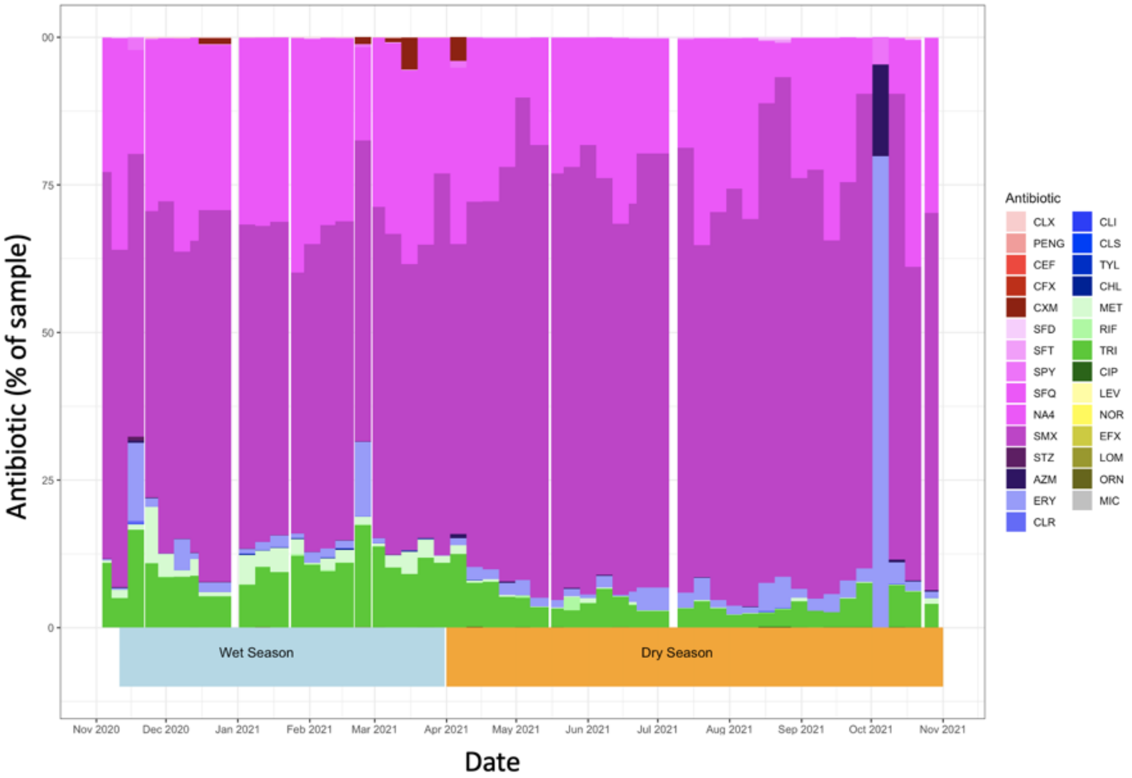

Figure S6. PEARSONS MATRIX OF ANTIBIOTICS IN RIVER WATER FROM URBAN STUDY SITES. Correlation coefficients are illustrated on a colour spectrum, with those in red and orange showing the highest degree of relationship.

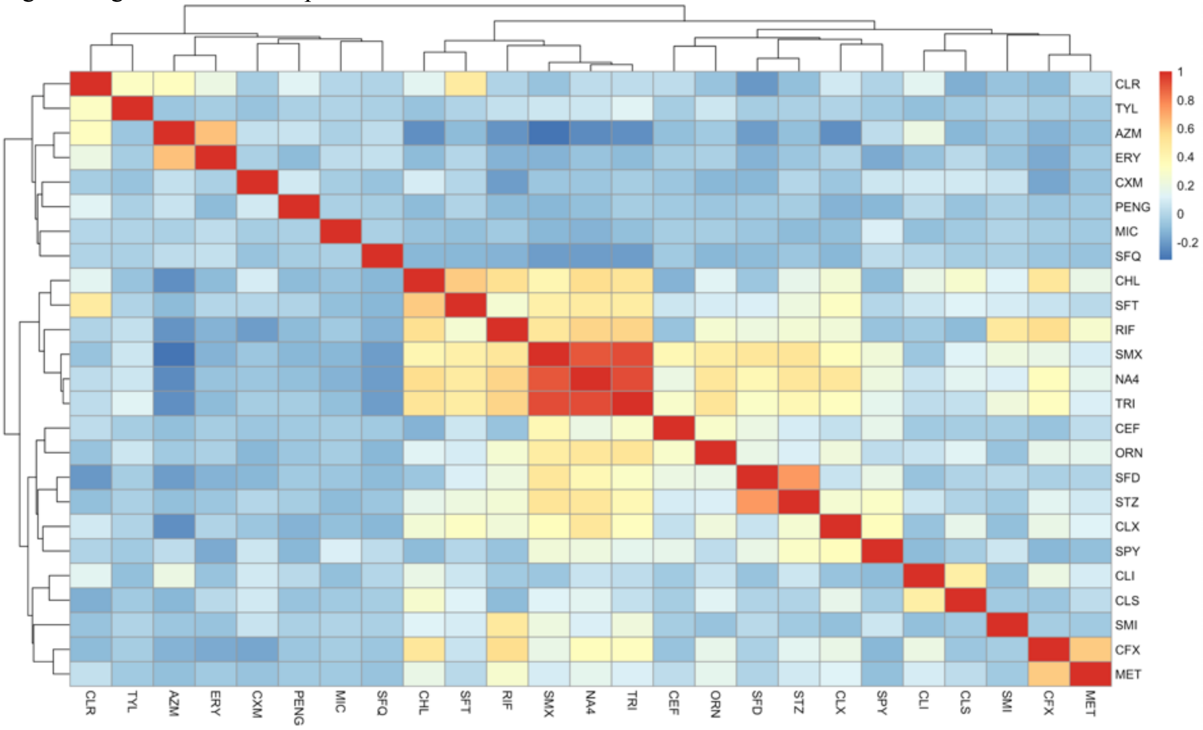

Figure S7a. TEMPORAL RELATIONSHIPS IN THE RECOVERY AND CONCENTRATIONS OF CUMULATIVE MACROLIDE RISK IN RIVER WATER, STRATIFIED BY SAFE AND UNSAFE PNEC LEVELS FOR EACH SITE.

Monthly trends in the presence and absence (white) of macrolides are plotted over a 1-year period, spanning across the wet (blue bar) and dry (yellow bar) season. These have been stratified into safe (green, <PNEC) and unsafe (red, >PNEC) levels based on the concentrations identified. Values inside the cells describe the ratio of analyte:PNEC illustrating the levels of risk. A value of 0 denotes where an antibiotic was identified above the LOQ but below 0.01% of the agreed PNEC target. To quantify a cumulative macrolide risk (ng/POCIS^{-1}/day^{-1}l) we have adjusted each macrolide antibiotic concentration (ng/POCIS^{-1}/day^{-1}) returned from a sampler compared to a relative PNEC concentrations and added these together, as per the below equation. This will then be compared to the PNEC of azithromycin (20ng/L) to determine if the cumulative risk has exceeded the PNEC level.

$$\begin{aligned} &\textit{Modelled cummulative Macrolide risk} \\ &\quad = \textit{azithromycin}\,(\textit{ng per litre}) + \textit{clarithromycin}\,\left(\frac{\textit{ng per litre}}{4}\right) + \textit{clindamycin}\,\left(\frac{\textit{ng per litre}}{5}\right) \\ &\quad + \textit{erythromycin}\,\left(\frac{\textit{ng pre litre}}{25}\right) \end{aligned}$$

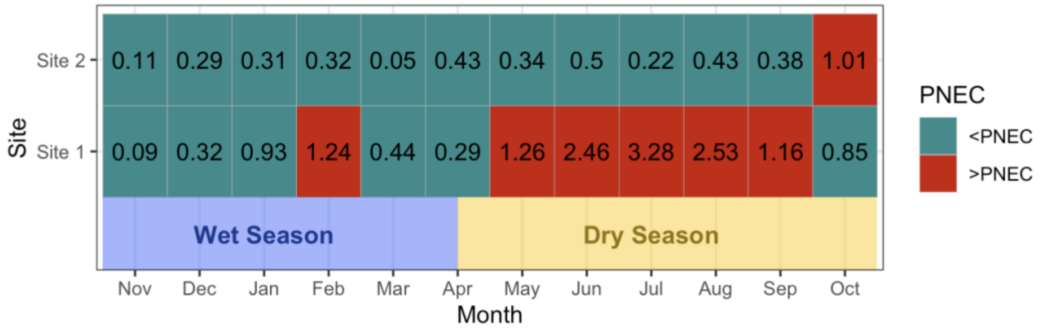

Figure S7b. **TEMPORAL RELATIONSHIPS IN THE RECOVERY AND CONCENTRATIONS OF CUMULATIVE FLUOROQUINOLONE RISK IN RIVER WATER, STRATIFIED BY SAFE AND UNSAFE PNEC LEVELS FOR EACH SITE.**

Monthly trends in the presence and absence (white) of fluoroquinolones are plotted over a 1-year period, spanning across the wet (blue bar) and dry (yellow bar) season. These have been stratified into safe (green, <PNEC) and unsafe (red, >PNEC) levels based on the concentrations identified. Values inside the cells describe the ratio of analyte:PNEC illustrating the levels of risk. A value of 0 denotes where an antibiotic was identified above the LOQ but below 0.01% of the agreed PNEC target. To quantify a cumulative fluoroquinolone risk (ng/POCIS^{-1}/day^{-1}) we adjusted each fluoroquinolone antibiotic concentration (ng/POCIS^{-1}/day^{-1}) returned from a sampler compared to a relative PNEC concentrations and added these together, as per the below equation. This will then be compared to the PNEC of ciprofloxacin (60ng/L) to determine if the cumulative risk has exceeded the PNEC level.

$$\begin{aligned} &\textit{Modelled cummulative FQ risk} \\ &= ciprofloxacin\,(ng\ per\ litre) + enrofloxacin\,(ng\ per\ litre) + \ flumequine\,\left(\frac{ng\ per\ litre}{4.17}\right) \\ &+ \ levofloxacin\,\left(\frac{ng\ per\ litre}{4.17}\right) + Norfloxacin\,\left(\frac{ng\ pre\ litre}{8.33}\right) \end{aligned}$$

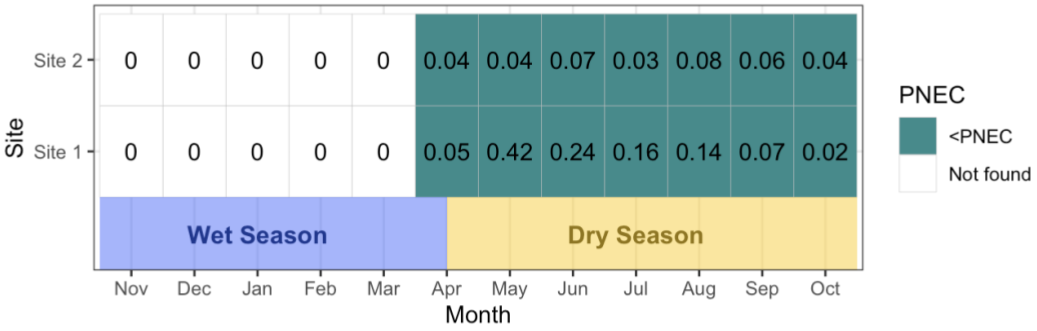

Figure S8a. SPATIOTEMPORAL VARIATIONS IN MEDICAL COMPOSITIONS FOUND AT SITE 1 OVER A 1-YEAR PERIOD. Presented as the percentage (%) of the total medication concentration normalised to sampling time (ng/POCIS^{-1}/day^{-1}) representing each medication.

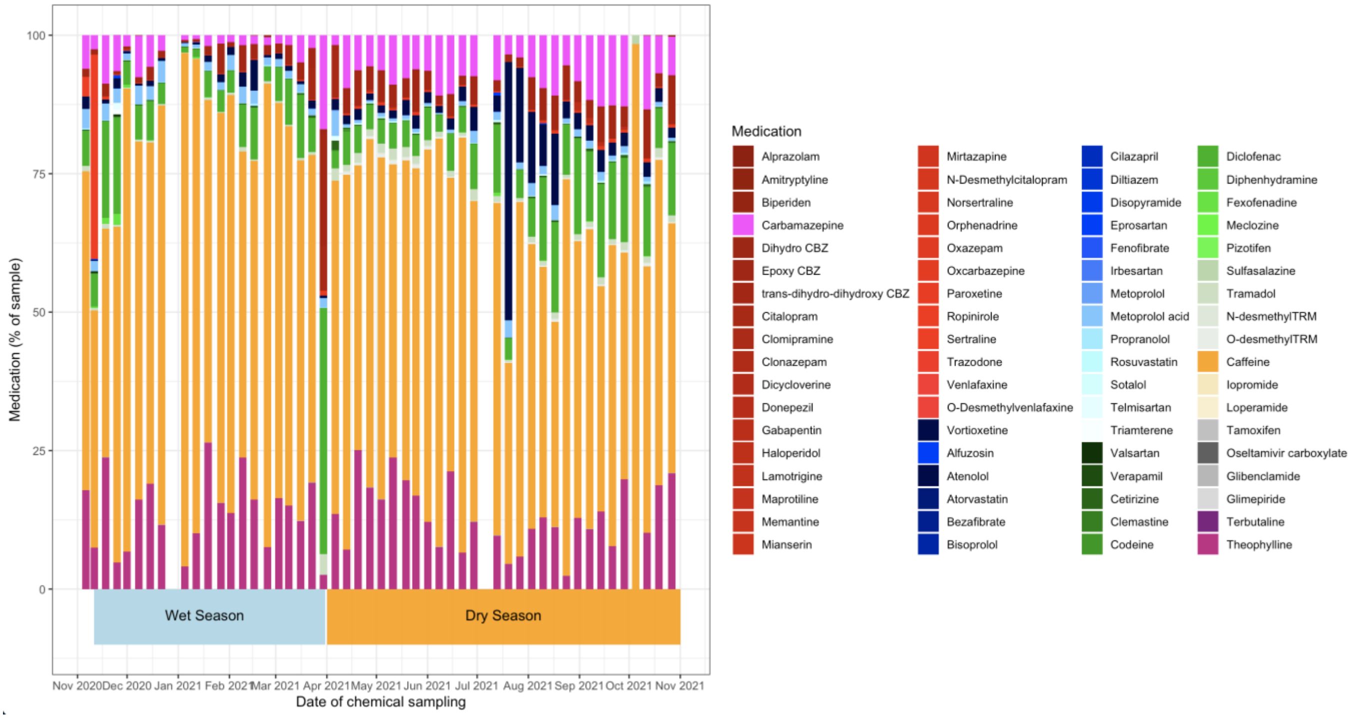

Figure S8b. SPATIOTEMPORAL VARIATIONS IN MEDICAL COMPOSITIONS FOUND AT SITE 2 OVER A 1-YEAR PERIOD. Presented as the percentage (%) of the total medication concentration normalised to sampling time (ng/POCIS^{-1}/day^{-1}), representing each medication.

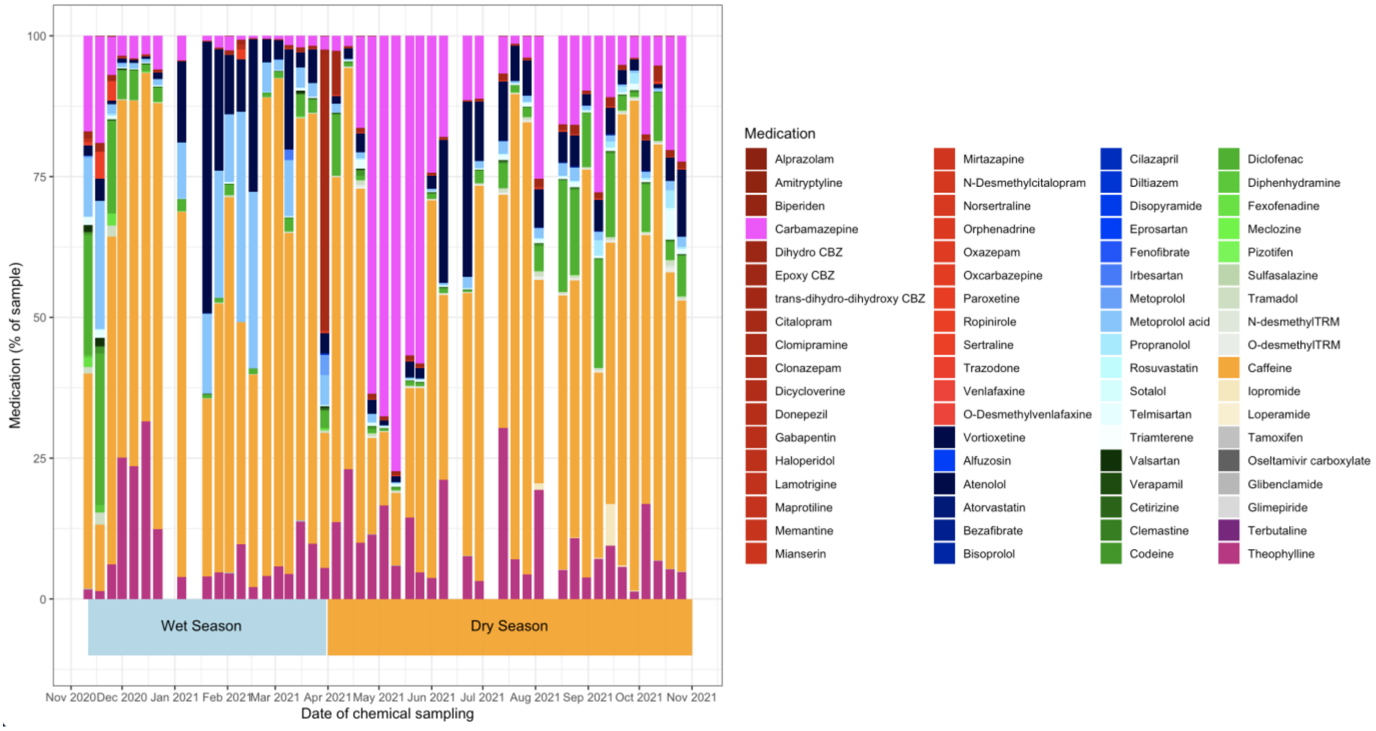

Figure S9a. CUMULATIVE TOTAL OF MEDICATIONS IDENTIFIED FROM SITE 1, NORMALISED TO SAMPLING TIME (ng/POCIS^{-1}/day^{-1}), STRATIFIED BY SITE AND COLOURED BY MEDICATION CLASS.

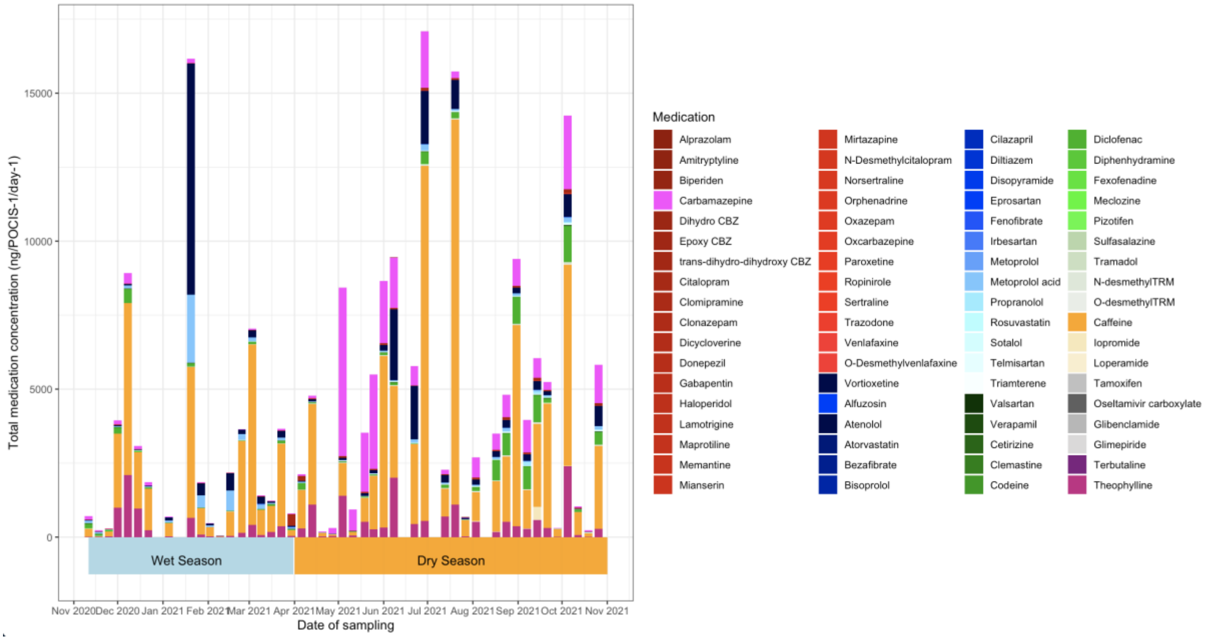

Figure 9b. CUMULATIVE TOTAL OF MEDICATIONS IDENTIFIED FROM SITE 2, NORMALISED TO SAMPLING TIME (ng/POCIS^{-1}/day^{-1}), STRATIFIED BY SITE AND COLOURED BY MEDICATION CLASS.

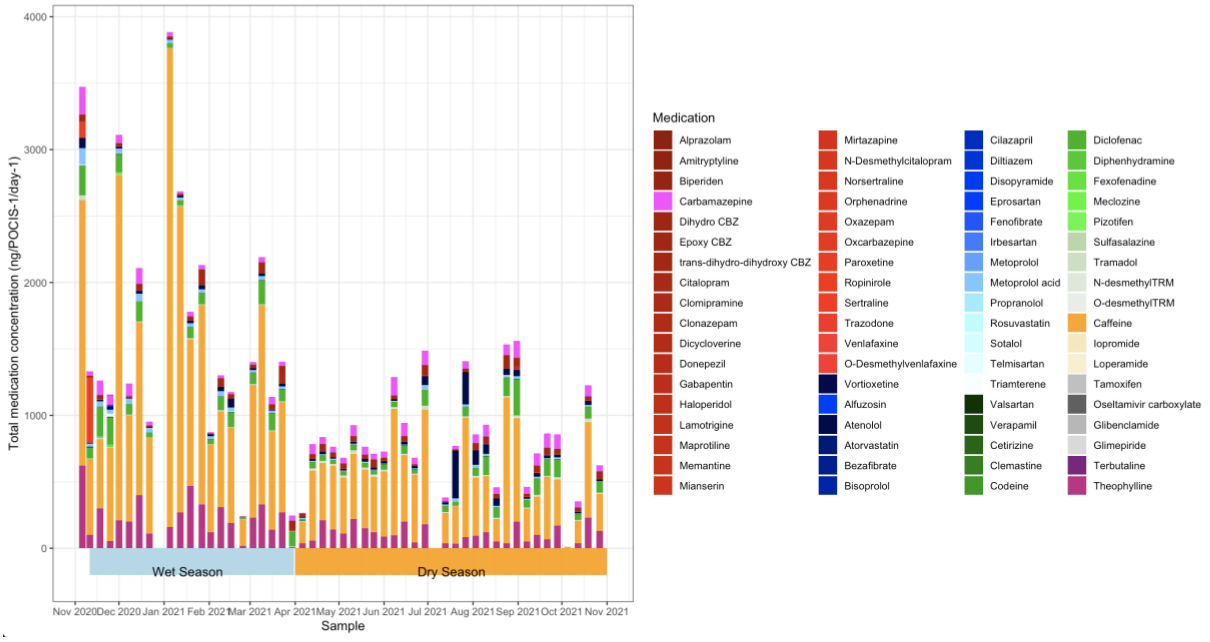

Figure S10a. TEMPORAL RELATIONSHIPS IN THE RECOVERY AND CONCENTRATION OF MEDICATIONS IN RIVER WATER FROM SITE 1, STRATIFIED INTO SAFE AND UNSAFE PNEC/CEC LEVELS.

Monthly trends in the presence and absence (white) of antibiotics are plotted over a 1-year period, spanning across the wet (blue bar) and dry (yellow bar) season. Medications have been stratified into safe (green, <PNEC/CEC) and unsafe (red, >PNEC/CEC) levels based on the concentrations identified. Values inside the cells describe the

ratio of analyte:PNEC/CEC illustrating the levels of risk. A value of 0 denotes where an antibiotic was identified above the LOQ but below 0.01% of the agreed PNEC/CEC target.

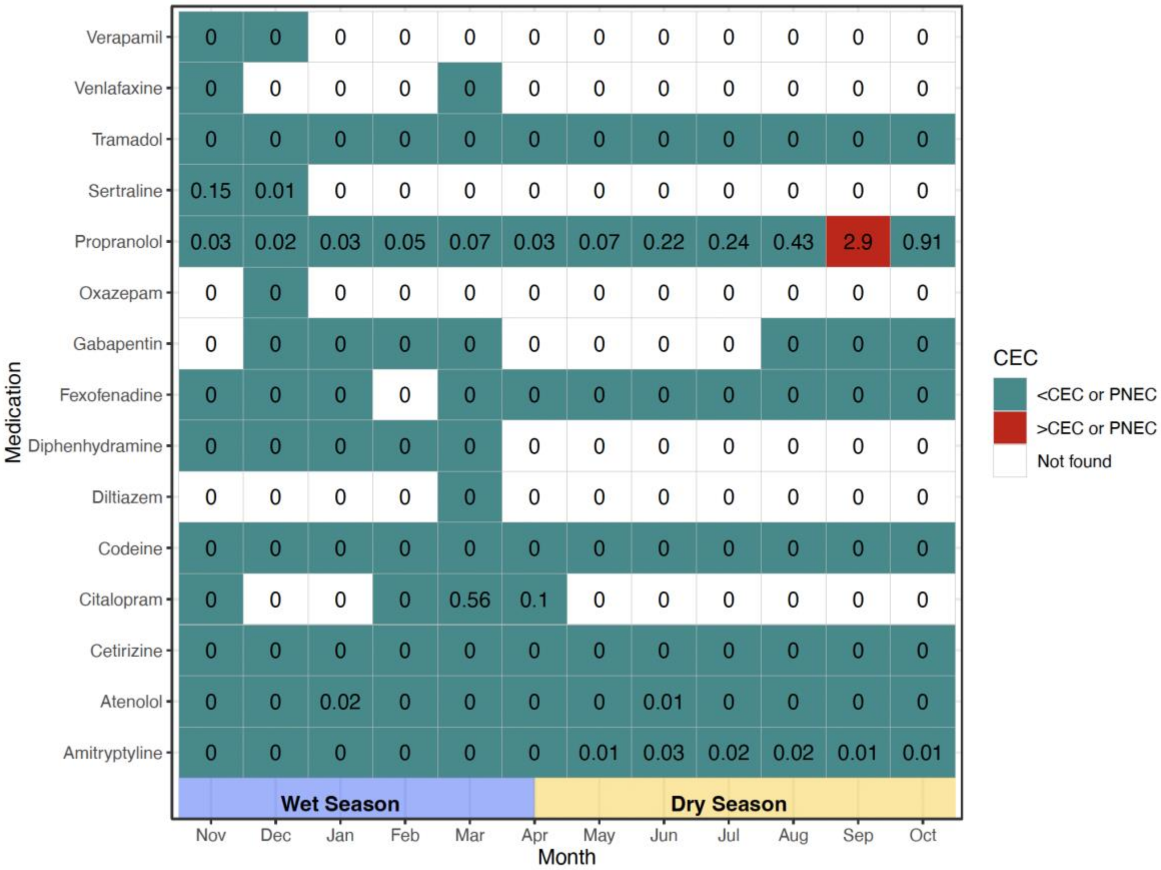

Figure S10b. TEMPORAL RELATIONSHIPS IN THE RECOVERY AND CONCENTRATION OF MEDICATIONS IN RIVER WATER FROM SITE 2, STRATIFIED INTO SAFE AND UNSAFE PNEC/ CEC LEVELS.

Monthly trends in the presence and absence (white) of antibiotics are plotted over a 1-year period, spanning across the wet (blue bar) and dry (yellow bar) season. Medications have been stratified into safe (green, <PNEC/CEC) and unsafe (red, >PNEC/CEC) levels based on the concentrations identified. Values inside the cells describe the ratio of analyte:PNEC/CEC illustrating the levels of risk. A value of 0 denotes where an antibiotic was identified above the LOQ but below 0.01% of the agreed PNEC/CEC target.

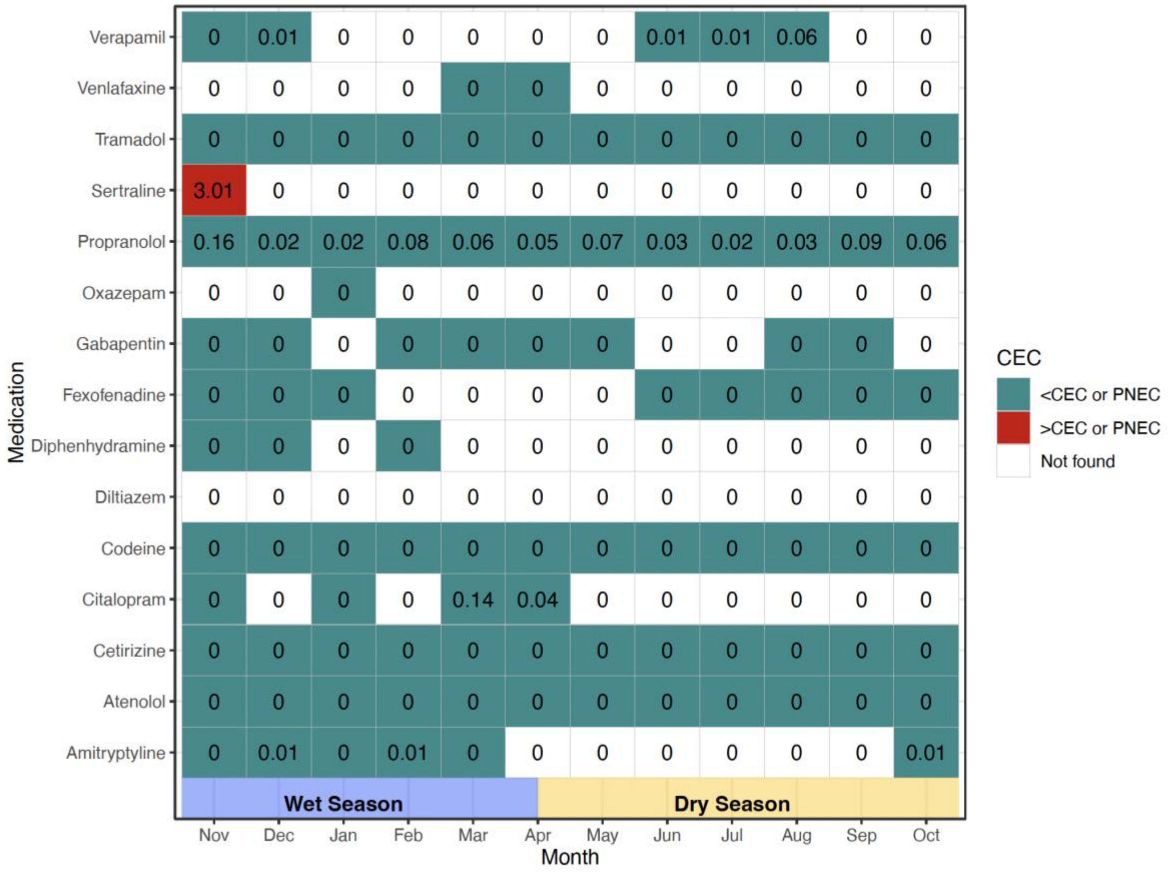

Figure S11. DETAILED MAPS OF THE RIVERINE NETWORK OF BLANTYRE, INCLUDING (a) BLANTYRE CITY (b) NDIRANDE, AND (c) CHILEKA.

DRUM study polygons have been demarcated in orange. Sampling sites have been geolocated (site 1: star, site 2: triangle, site 3: square, site 4: circle, site 5: diamond) alongside the key rivers (black = Mudi river, red = Nasolo river, blue = unnamed river).

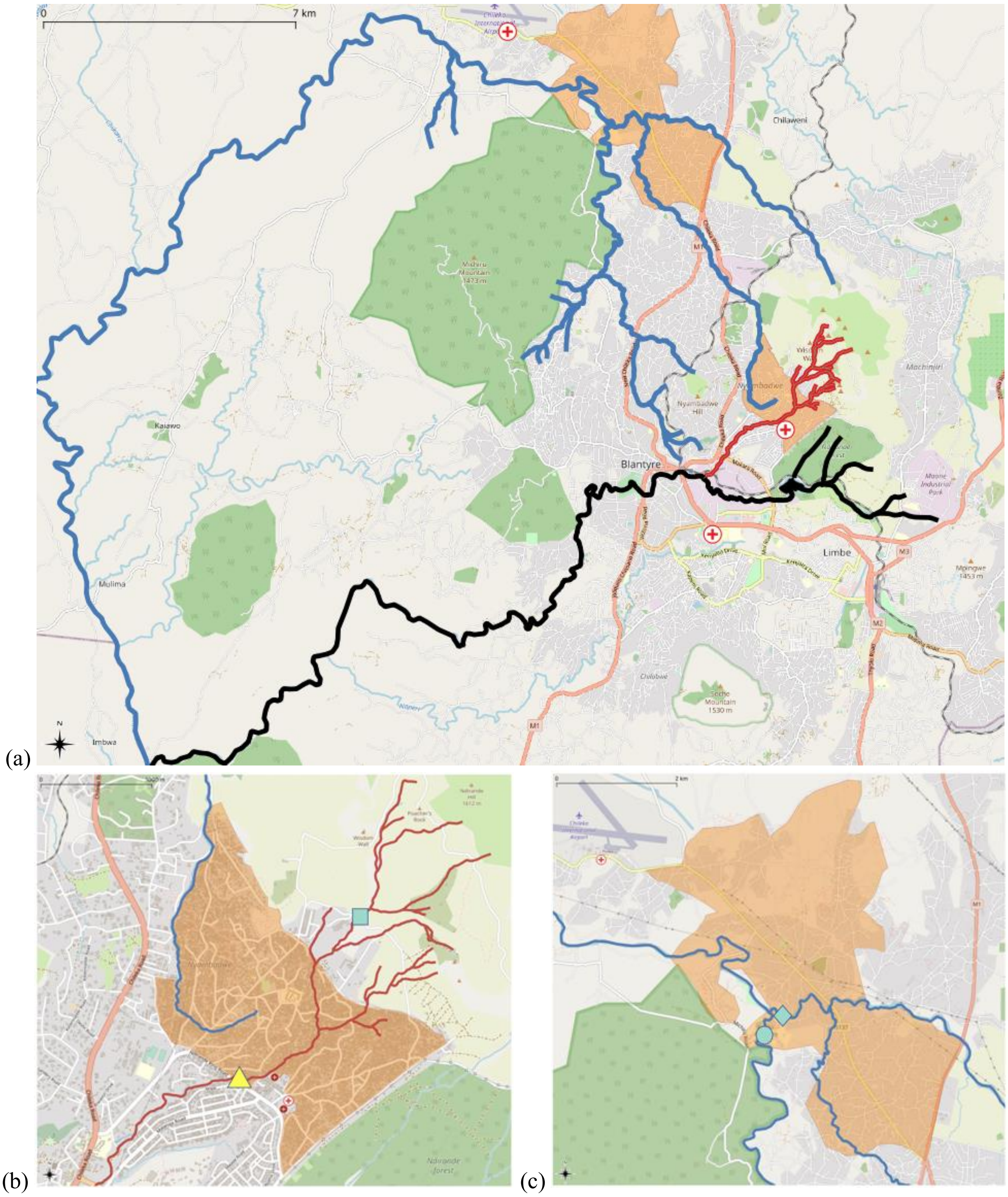

Figure S12. PHOTOS OF THE SAMPLING LOCATIONS, INCLUDING BOTH STUDY (1&2) AND PILOT (3,4 &5) SITES. Local approvals and permissions were granted.

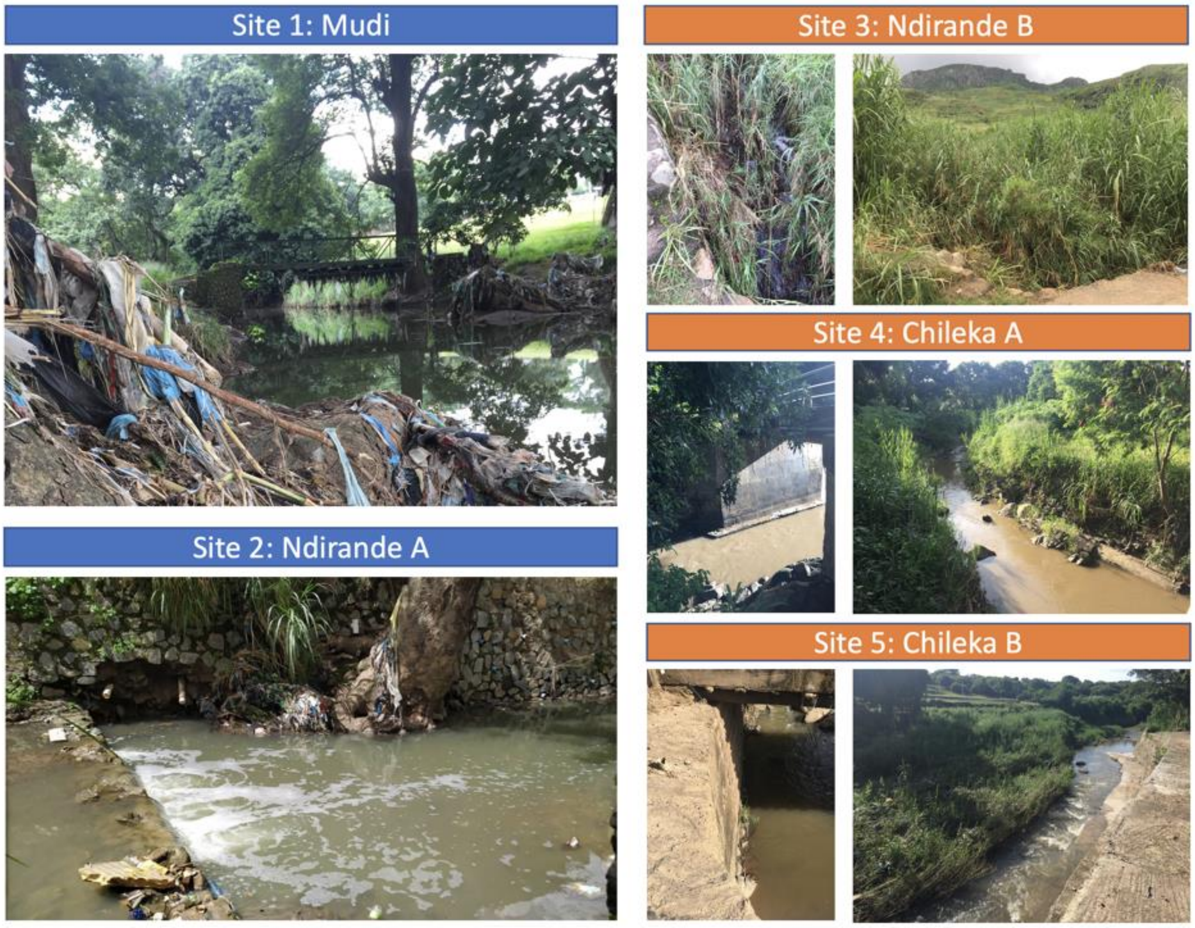

Figure S13. SEASONAL CHANGES IN THE RIVERS AT SAMPLING SITES. Photos were taken during the pilot and continuous phase, after approvals and local permissions were granted.

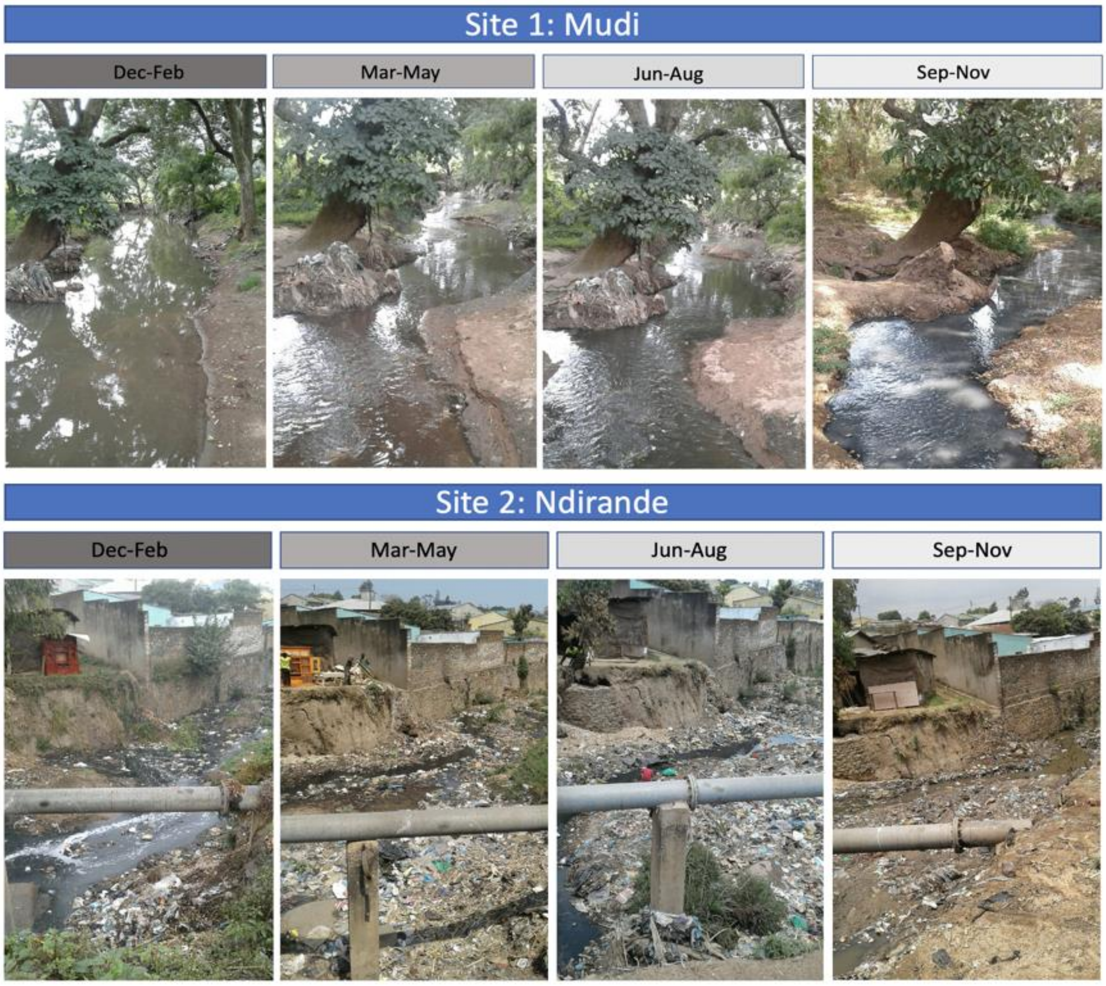

Figure S14. POCIS SAMPLER
Porous metal cage (a) sandwiches the PES membrane (b) allowing for environmental exposure while protecting the membrane integrity, which is attached to a metal wire that is secured to the river bank (c).

a) outside cage b) internal PES membrane. c) completed POCIS with wire

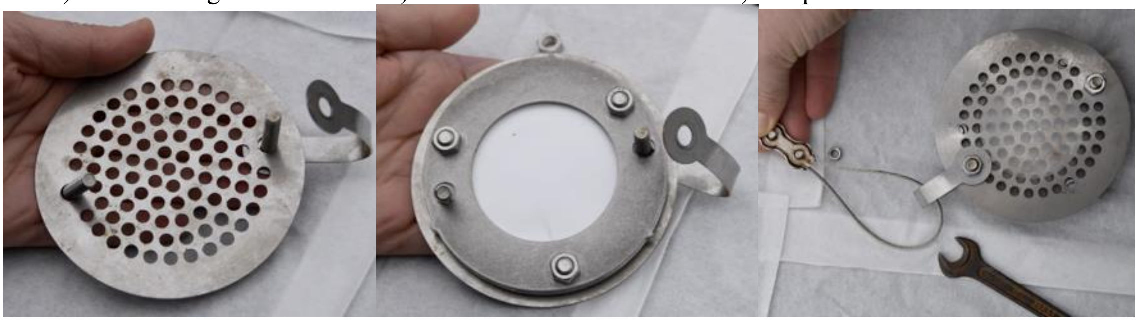

Pictures taken from reference 24 (*Guide for the installation of POCIS passive sampler. Faculty of Fisheries and Protection of Waters, University of South Bohemia, Czech Republic*).
